# Integrating Spatial Transcriptomics and Single-Cell RNA-seq Reveals the Gene Expression Profling of the Human Embryonic Liver

**DOI:** 10.3389/fcell.2021.652408

**Published:** 2021-05-20

**Authors:** Xianliang Hou, Yane Yang, Ping Li, Zhipeng Zeng, Wenlong Hu, Ruilian Zhe, Xinqiong Liu, Donge Tang, Minglin Ou, Yong Dai

**Affiliations:** ^1^Department of Clinical Medical Research Center, Guangdong Provincial Engineering Research Center of Autoimmune Disease Precision Medicine, The Second Clinical Medical College, Jinan University (Shenzhen People’s Hospital), Shenzhen, China; ^2^The First Affiliated Hospital, Jinan University, Guangzhou, China; ^3^Shenzhen Far-East Women & Children Hospital, Shenzhen, China; ^4^Central Laboratory, Guangxi Health Commission Key Laboratory of Glucose and Lipid Metabolism Disorders, The Second Affiliated Hospital of Guilin Medical University, Guilin, China; ^5^Guangxi Key Laboratory of Metabolic Disease Research, Central Laboratory, Nephrology Department of Guilin No. 924 Hospital, Guilin, China

**Keywords:** spatial transcriptomics, single-cell RNA sequencing, multimodal intersection analysis, fetal liver, hepatoblast, erythrocyte

## Abstract

The liver is one of vital organs of the human body, and it plays an important role in the metabolism and detoxification. Moreover, fetal liver is one of the hematopoietic places during ontogeny. Understanding how this complex organ develops during embryogenesis will yield insights into how functional liver replacement tissue can be engineered and how liver regeneration can be promoted. Here, we combine the advantages of single-cell RNA sequencing and Spatial Transcriptomics (ST) technology for unbiased analysis of fetal livers over developmental time from 8 post-conception weeks (PCW) and 17 PCW in humans. We systematically identified nine cell types, and defined the developmental pathways of the major cell types. The results showed that human fetal livers experienced blood rapid growth and immigration during the period studied in our experiments, and identified the differentially expressed genes, and metabolic changes in the developmental process of erythroid cells. In addition, we focus on the expression of liver disease related genes, and found that 17 genes published and linked to liver disease mainly expressed in megakaryocyte and endothelial, hardly expressed in any other cell types. Together, our findings provide a comprehensive and clear understanding of the differentiation processes of all main cell types in the human fetal livers, which may provide reference data and information for liver disease treatment and liver regeneration.

## Introduction

The liver consists of greater than 20 cell types, including hepatocytes, liver endothelial cells, biliary ductal cells (cholangiocytes), mesothelial cells, Kupffer cells, and various circulatory immune cells, which are all organized to form the foundation for liver functions, including glycolytic and urea metabolism, immune responses, and drug detoxification (Wang et al., 2020). In the development of human fetus, liver is an essential hemopoietic organ. Hematopoietic stem and progenitor cells (HSPCs) immigrated into the liver bud at approximately W6 in humans (Ivanovs et al., 2017; Gao and Liu, 2018). Then, the fetal liver becomes the major hematopoietic organ and provides a specific niche for HSPC proliferation and differentiation (Golub and Cumano, 2013). The liver is one of the vital organs of the human body, which vulnerable to a variety of pathogenic factors inside and outside the body, causing inflammation and damage. It is perhaps not surprising that liver diseases are major contributors to morbidity and mortality (Gordillo et al., 2015). Besides, the liver has a strong regenerative capacity. After partial hepatectomy, liver regeneration depends on the regenerative capacity of the residual liver tissue, which has aroused broad public concern. Therefore, a comparative study of fetal liver development is also vital for our understanding of liver development and regeneration mechanisms.

The rapid advances in massively parallel DNA sequencing during the past decade have enabled a view into liver development at unprecedented molecular resolution. Bulk cell RNA-sequencing (RNA-seq) technology has made it possible to obtain unbiased high-throughput gene expression data from bulk tissue and individual cells (Salmen et al., 2018). However, conventional RNA-seq methods process millions of cells, and cellular heterogeneity cannot be addressed because signals of variably expressed genes would be averaged across cells (Hou et al., 2016). Understanding biological systems require knowledge of their components. Although single-cell RNA-sequencing (scRNA-seq) is a powerful tool for addressing transcriptional heterogeneity (Moncada et al., 2020). The application of such single-cell analysis has greatly facilitated experimental studies in stem cell properties (van Wolfswinkel et al., 2014), cellular immunity (Shalek et al., 2013), cancer diagnosis (Dalerba et al., 2011), and developmental processes (Xue et al., 2013). However, tissue dissociation before sequencing results in the loss of the cells’ positional information, thus limiting our understanding of cellular organization and interactions in the fetal liver development (Moncada et al., 2020). To overcome these deficiencies, Spatial Transcriptomics (ST) allows for the spatial mapping of RNA-seq transcript data onto high-resolution tissue images, with the spatial information maintained by using a unique microarray composed of >1000 spots (100-μm circular areas) with barcoded capture probes (Wong et al., 2018). However, the lack of cellular resolution is the main limitation of ST. The 100-μm spatial spots, with a center-to-center spacing of 200 μm, typically cover 5–100 cells each, depending on region and tissue type. Therefore, this resolution currently prevents analysis at the single-cell level (Salmen et al., 2018). To overcome this limitation, the applications of some new analytical methods in ST were able to identify cell types more accurately (Moncada et al., 2020).

In this study, we used an integration of scRNA-seq with the ST technology to perform unbiased analysis of fetal livers over developmental time from W8 and W17 in humans. Multimodal intersection analysis (MIA) was applied to integrate scRNA-seq and ST datasets (Moncada et al., 2020). We systematically identified nine cell types, as well as various specific clusters within each cell types. We also defined cell lineage differentiation pathways in human fetal livers. Moreover, we observed significant differences in cell composition, cell heterogeneity, and gene expression during human fetal liver development. It is worth noticing that we found that 17 genes published and linked to liver disease mainly expressed in megakaryocyte and endothelial, hardly expressed in any other cell types. In summary, we present a spatiotemporal atlas that comprehensively and systematically describes the cellular heterogeneity and spatial archetypes of the developing human liver, facilitating our understanding of disease origins, and will help *de novo* generation of liver cell types and liver structures.

## Materials and Methods

### Biological Materials

Two human developmental liver tissues were used in the study. Clinical age and post-conceptional were determined using clinical ultrasound and stage-dependent anatomical landmarks of the embryos: 8 post-conception weeks (PCW) and 17 PCW. Samples were collected after elective surgical abortions at Shenzhen People’s Hospital. Written informed consent was obtained before sample collection. This study was conducted following the tenets of the Declaration of Helsinki and was approved by the Ethics Committee of the Shenzhen People’s Hospital, China (ref. no. LL-KY-2019591). The tissue samples were snap-frozen in isopentane pre-chilled with liquid nitrogen and stored at −80°C until sectioning.

### Tissue Staining and Imaging

Liver tissue was gently washed with cold PBS and snap-frozen in isopentane (2-methyl butane, Sigma) on dry ice. The tissue was subsequently embedded in Tissue-Tek (OCT) and snap-frozen using an isopentane/dry ice slurry (Asp et al., 2017). The liver tissues were cryosectioned at 10 μm thickness and systematically placed on chilled Visium Tissue Optimization Slides (3000394, 10× Genomics) and Visium Spatial Gene Expression Slides (2000233, 10× Genomics), and stored at −80°C until use. Here’s the information of superfrost microscope glass slides: Each of the spots printed onto the array was 200 μm from the center to center, and 100 μm in diameter, covering an area of 6,200 × 6,600 μm (Moncada et al., 2020). Spots were printed with approximately 2 × 10^8^ oligonucleotides containing a randomized 7-mer unique molecular identifier (UMI), an 18-mer spatial barcode, and a poly-20TVN transcript capture region. For processing, the tissues were first warmed to 37°C for 1 min and fixed in 36.5% formaldehyde (#F8775, Sigma-Aldrich) diluted 1:10 in 1 × PBS (#09-9400, Medicago) for 10 min and then washed in 1 × PBS. Next, the tissues were dehydrated with isopropanol for 1 min followed by staining with H&E. Slides were mounted in 80% glycerol, and brightfield images were taken on a 10× objective (Plan APO) on a Nikon Eclipse Ti2-E (27755 × 50783 pixels for TO, 13332 × 13332 pixels for GEX).

### Permeabilization and Reverse Transcription

After brightfield imaging, exonuclease pre-permeabilization was performed with 0.2 mg ml-1 BSA and 200 units of collagenase diluted in HBSS buffer at 37°C for 20 min and washed with 100 μl of 0.1 × SSC buffer. Tissue was permeabilized with 0.1% pepsin in HCl at 42°C for 4 min and washed with 100 μl of 0.1 × SSC buffer. Next, RT Master Mix containing reverse transcription reagents [1 × First strand buffer (Invitrogen), 0.5 mM of each dNTP, 5 mM dithiothreitol, 0.2 μg μl-1 BSA, 1% dimethylsulfoxide, 50 ng μl-1 Actinomycin D, 2 U μl-1 RNaseOUT (Invitrogen) and 20 U μl-1 Superscript III (Invitrogen)] was added to the permeabilized tissue sections, and incubated at 42°C overnight (∼17 h). Tissues were then digested away from the slide by incubating the tissue with 1% 2-mercaptoethanol in RLT buffer (Qiagen) at 56°C for 1 h with continuous shaking, then incubation in proteinase K (Qiagen) diluted 1:8 in PKD buffer (Qiagen) for 1 h at 56°C with continuous shaking (Asp et al., 2017). After RT and tissue removal (the cDNA is not cleaved), the slides were imaged to visualize the incorporated fluorescent nucleotides, together creating a cDNA footprint. The optimal permeabilization conditions depended on these images (Berglund et al., 2018). The assessment was based on how diffused the fluorescent print and the signal intensity compared with the tissue morphology. Optimal conditions would produce a strong and morphologically correct fluorescent print. Here, we selected 6 min as the optimal time based on tissue optimization time course experiments.

### Release of Probes, ST Library Preparation and Sequencing

To enable sharp, spatially barcoded experiments after RT and tissue removal, the cDNA was enzymatically cleaved from the surface and collected in tubes ready for downstream library generation. The cDNA release step was done at 37°C for 1 h and 15 min. Once the surface probes were de-attached, 65 μl from each well was collected. After probe release, the array was imaged once more with a hybridized fluorescent probe to create a spatial spot image, as previously described (Salmen et al., 2018). The released cDNA was converted into dsDNA, using the hybridized RNA as the primer. Afterward, the dsDNA was then purified using beads and underwent *in vitro* transcription (IVT) overnight, which mixture contained 1 × T7 Enzyme Mix (Ambion, AM1334), 1 × T7 Reaction Buffer (Ambion, AM1334), 7.5 mM of each NTP (Ambion, AM1334), and 1 U/μl SUPERaseIN (Ambion, AM2694). The remaining purified cDNA was indexed using the following program: 98°C for 3 min, followed by 25 cycles of 98°C for 20 s, 60°C for 30 s, and 72°C for 5 min. The concentrations were measured using a Qubit dsDNA HS Assay Kit (Invitrogen), purified libraries were assessed using a 2100 Bioanalyzer (Agilent) according to the manufacturer’s instructions (Moncada et al., 2020). The finished libraries were sequenced on the Hiseq3000 System (Illumina), and the sequencing depth of each sample was approximately 250–270 M read-pairs. The following read protocol was performed: read 1, 28 cycles; i7 index read, 10 cycles; i5 index read, 10 cycles; read 2, 91 cycles.

### ST Raw Data Annotation, Filtering, and Processing

Histology images and raw FASTQ files were processed by sample with the Space Ranger software (version 1.0.0), which used STAR v.2.5.1b for genome alignment (Dobin et al., 2013) against hg38 reference genome “refdata-gex-GRCh38-2020-A.” Briefly, read 2 was mapped against the reference GRCh38 human genome, and read 1 was used for spatial information and UMI filtering. Data were demultiplexed based on the spatial barcodes, and duplicate reads (generated through amplification) were removed by UMI filtering. FASTQ files containing the gene count data for each spatial barcode were produced. Using Bowtie2 (version 2.3.1) to align the demultiplexed FASTQ files (Langmead and Salzberg, 2012), and using HTSeq (version 0.9.1) to count UMIs (Anders et al., 2015). We obtained information about the number of spots under the tissue, median genes per spot, the sum of UMIs per spot, etc. Detailed information on sequencing data processing is available in a recently published study (Stahl et al., 2016). First, spots with fewer than 200 genes were filtered. UMI counts in each spot were normalized by the total transcript count and then scaled by the median number transcript count across all spots. Besides, log2 transformed gene expression data was used. Spots were then clustered with hierarchical clustering based on the top variably expressed genes. The approach was performed using the Seurat package (version 3.1.5)^1^ (Satija et al., 2015). To identify differentially expressed genes (DEGs), pair-wise comparisons of individual clusters against all other clusters were implemented using the FindAllMarkers function (settings: logfc.threshold = 0.1, test.use = “bimod,” min.pct = 0.01) in the Seurat package. Visualization of ST clusters was carried out using Uniform Manifold Approximation and Projection (UMAP) or t-Distributed Stochastic Neighbor Embedding (TSNE) in the Seurat package.

### Cell Identification by ScRNA-seq and Determination of Cell Type by Multimodal Intersection Analysis (MIA)

In this study, the following publically available scRNA-seq datasets used were as follows. KeenEye Technologies-hosted Platform accession numbers: FCAImmP7277552, FCAImmP7277553, FCAImmP7352192, FCAImmP7352193, FCAImmP7352194, and FCAImmP7352195. All raw files are available at https://www.ebi.ac.uk/arrayexpress/experiments/E-MTAB-7407/. The data processing and statistical analysis were performed as Popescu et al. (2019) described. Briefly, Cell Ranger Single-Cell Software Suite (version 2.0.2, 10× Genomics Inc.) was utilized to align and quantify the sequencing data using the GRCh38 human reference genome (official Cell Ranger reference, version 1.2.0). htseq-count (version 0.10.0) was utilized to calculate gene-specific read counts. Cells with fewer than 200 detected genes and for which the total mitochondrial gene expression exceeded 20% were removed. Genes that were expressed in fewer than three cells were also removed. Cluster cell identity was assigned by manual annotation using known marker genes. Subsequently, we integrated our ST data and these publically available scRNA-seq data by introducing MIA as previously reported (Moncada et al., 2020). The hypergeometric cumulative distribution was used to identify the significance of the overlap between cell type marker genes and ST genes, with all genes as the background to compute the *P* value.

### Advanced Analysis of Cell State and Function Based on R Package

ClusterProfiler R package was used for Gene Ontology (GO) characteristics and KEGG enrichment. Monocle was used for the developmental trajectory visualization. Weighted gene co-expression network analysis (WGCNA) algorithm was used to perform cross-cluster comparison and weighted correlation network analysis. Briefly, we screened differential expressed gene sets in the each cell clusters and constructed a complex gene co-expression network. Subsequently, WGCNA identified essential modules which were consisted of highly interconnected genes using unsupervised clustering. Moreover, cellular communication and signaling transduction regulation between cell clusters (or cell type) was performed using circlize package. In addition, we performed the cell cycle analyses with core gene set as previously reported (Fan et al., 2018), which including 54 G2/M genes and 43 G1/S. The average expression of each gene set was calculated as the corresponding score. If their G2/M score <2 and G1/S score <2, cells were determined to be quiescent. Otherwise, they were deemed proliferative. Moreover, if their G1/S score < G2/M score, proliferative cells were designated G2/M; If their G2/M score < G1/S score, cells were designated G1/S.

## Results

### Global Clustering and Identification of Cell Type

**FIGURE 1:**
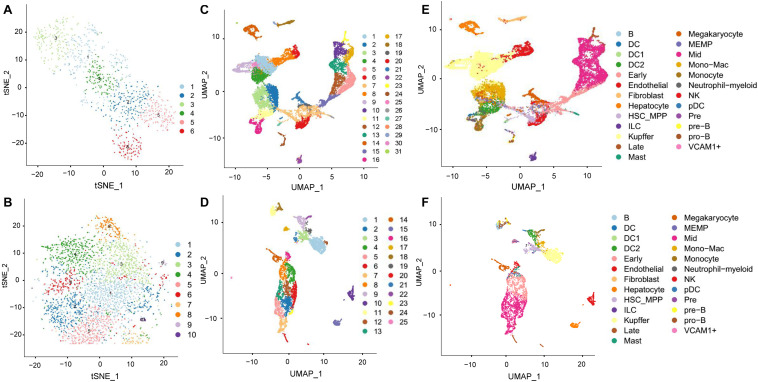
ST and scRNA-seq identified major cell types in the human fetal livers. **(A,B)** The t-SNE plot of all spots from ST data of 8 PCW liver **(A)** and 17 PCW liver **(B)**. Each point represents a spot, colored by cell types. **(C–F)** The UMAP plot of all cells from single-cell RNA-seq of 8 PCW liver **(C,E)** and 17 PCW liver **(D,F)**. Each point represents a cell, colored by their associated cluster **(C,D)**, and colored by cell types **(E,F)**.

To investigate immune cell and red blood cell development in the fetal liver, we used ST technology to dissect global spatiotemporal gene expression dynamics during human liver development. We collected embryonic livers at two-time points (8 PCW and 17 PCW). After filtering data, we detected 1,267 high-quality spots in 8 PCW liver, and on average, each spot contained 204,628 clean reads, 1,132 detected genes, and 5,924 UMIs. In the 17 PCW, the livers consisted of a total pool of 3,489 individual spots with an average of 78,576 reads, 8,829 UMIs, and 2,266 genes per spot after filtering (Supplementary Figure 1). The percentage of UMIs derived from mitochondrial and ribosomal genomes in each spots was displayed in Supplementary Figure 2. Through dimensionality reduction and clustering the spots of each ST array, we identified 6 and 10 major cell clusters in the 8 PCW liver (Figure 1A) and 17 PCW liver (Figure 1B) based on gene expression pattern, respectively. Owing to the lack of cellular resolution of ST, we used scRNA-seq to explore the gene expression heterogeneity of the 8 PCW and17 PCW live tissue. After applying quality control filters and normalization, the scRNA-seq data consisted of 25,186 cells and 9,153 cells in the 8 and 17 PCW liver, respectively, with a median of 9,239 and 11,248 unique UMIs and 2,454 and 2,705 uniquely expressed genes per cell. To explore the cell composition of live organ, we processed the single-cell data, and identified 31 and 25 cell states in the 8 and 17 PCW liver, each of which was annotated according to well-known marker genes from the literature (Figures 1C–F and Supplementary Figure 3). We designated these clusters as B cell, dendritic cell (DC), DC1, DC2, early erythroblast, endothelial, HSC_MPP, fibroblast, hepatocyte, kupffer, ILC, late erythroblast, MEMP, mast cell, megakaryocyte, mid erythroblast, monocyte, mono-mac, neutrophil-myeloid, NK, pDC, Pre, Pre-B, Pro-B, and VCAM1^+^.

### MIA of scRNA-seq and ST Data

To integrate the scRNA-seq and ST datasets, we applied MIA as previously reported (Moncada et al., 2020). This analysis proceeds by first delineating sets of tissue region-specific and cell-type-specific genes and then determining whether their overlap is lower (depletion) or higher (enrichment) than expected by chance. In the ST and scRNA-seq data, we identified the gene sets with significantly higher expression in each spatial region (or each cell type) relative to the others. With the gene sets extracted across the ST and scRNA-seq, MIA next computed the overlap between each pair of region-specific and cell type-specific gene sets and assessed significant enrichment or depletion via a hypergeometric test.

**FIGURE 2:**
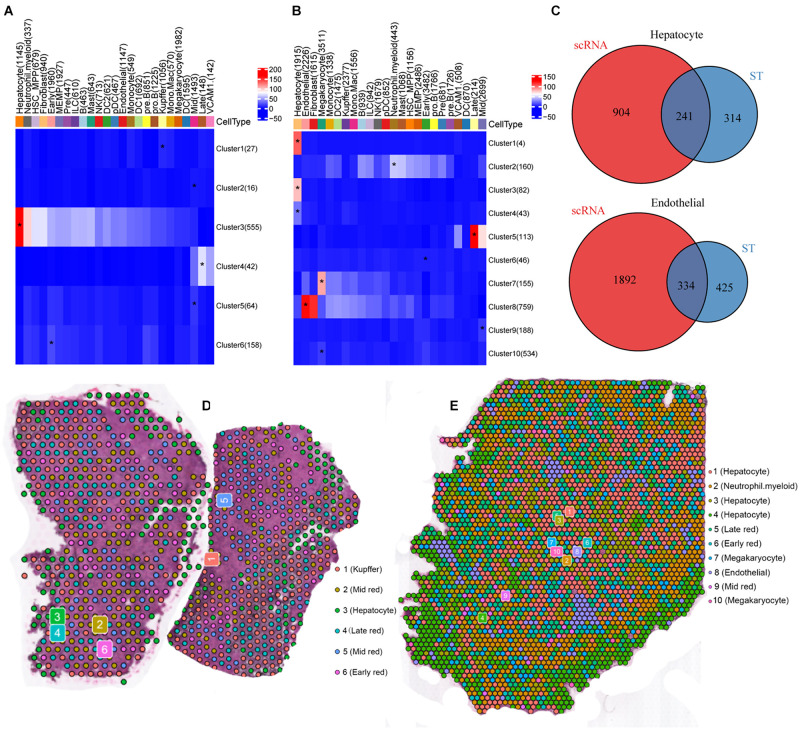
Spatially resolved gene expression of embryonic livers. **(A,B)** The MIA map of all scRNA-seq-identified cell types and ST-defined regions in 8 PCW **(A)** and 17 PCW liver **(B)**. The numbers of cell type- and tissue region-specific genes used in the calculation are shown. Red indicates enrichment (significantly high overlap); blue indicates depletion (significantly low overlap). **(C)** Venn diagram showing the overlaps of specifically expressed genes of hepatocyte and Endothelial cells between ST and scRNA-seq data. **(D,E)** The spots visualized at their original positions in the 8 PCW live tissue **(D)**, and the 17 PCW live tissue **(E)**. The colors indicate the clusters each spot is assigned to in the t-SNE plot in Figures 1A,B.

**FIGURE 3:**
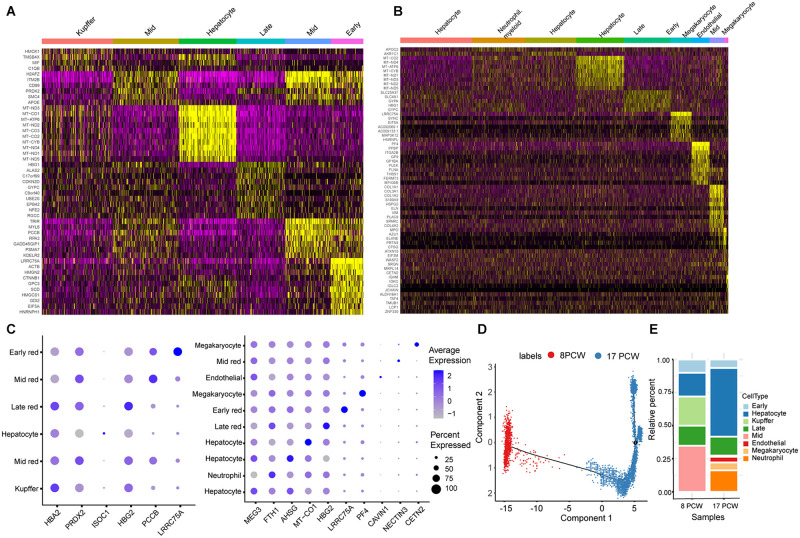
The specificity of marker gene expression. **(A,B)** Heatmap of standardized expression for the top 10 marker genes identified for each cell type in the 8 PCW **(A)** and 17 PCW liver **(B)**. Each row represents a signature gene, and each column represents a cell. Yellow indicates high expression of a particular gene, and purple indicates low expression. **(C)** The dot plot shows the relative expression of signature genes in each cell type of the 8 PCW (Left) and 17 PCW liver (Right). Gene-expression frequency (percentage of cells within each cell type expressing the gene) is indicated by spot size, and expression level is indicated by color intensity. **(D)** Pseudotime analysis shows the differentiation and relation of all spots in 8 PCW and 17 PCW live tissue. **(E)** Plot showing the proportional contribution of cell types identified in panel **(E)** to the fetal liver tissue over developmental time. PCW, post-conception weeks.

We found that the MIA approach was robust at identifying depletions and enrichments of cell types across spatial regions (Figures 2A,B). As an example, we found that the high expression genes of hepatocyte, kupffer cell, endothelial cell, and megakaryocyte overlapped significantly between the ST and scRNA-seq data (Figure 2C and Supplementary Figure 4). The 8 PCW liver was composed of kupffer, mid erythroblast, hepatocyte, late erythroblast, early erythroblast (Figure 2D). The 17 PCW liver was composed of hepatocyte, neutrophil, early erythroblast, mid erythroblast, late erythroblast, megakaryocyte, endothelial, and megakaryocyte (Figure 2E). Based on the average log fold-change, a heatmap of the top 10 genes unique to each cluster showed a high degree of heterogeneity between the clusters (Figures 3A,B). Besides, we choose one of the most distinct signature genes in each cell type, and presented as a dot plot to compare the differences (Figure 3C). Monocle was used to organize cells in a predicted developmental trajectory (pseudotime) according to transcriptional similarities. It yielded one tightly connected differentiation trajectory separated into two main branches corresponding to the different periods 8 and 17 PCW (Figure 3D).

### Spatially Resolved Heterogeneity of Human Fetal Hepatocytes

**FIGURE 4:**
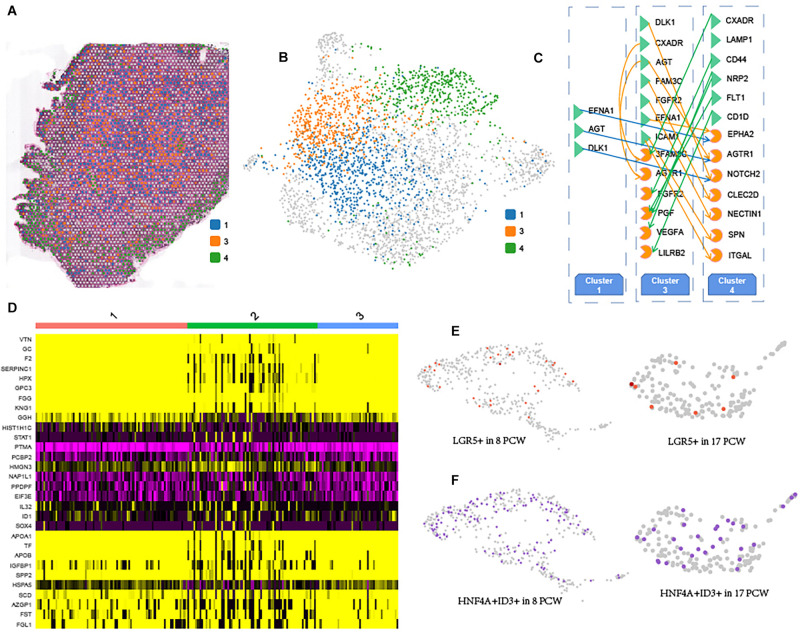
Spatially Resolved Heterogeneity of Human Fetal Hepatocytes. Three main subpopulations of hepatocyte were showed in tissue sections **(A)** and UMAP plot **(B)**. Matching plots showing the significant signaling transduction regulation between the Cluster 1-3-4 cells **(C)**. “Green triangle” represents the ligand, and “Orange irregular shape” represents receptor in the ligand-receptor pairs. Heat map of the top 10 abundant genes in each cluster **(D)**. Plots showing the expression of LGR5 in the hepatocyte of 8 PCW and 17 PCW liver **(E)**, and Plots showing the expression of HNF4A and ID3 gene in the hepatocyte of 8 PCW and 17 PCW liver **(F)**, and the gradient of red (purple) reflects expression levels.

As shown in Figure 3E, hepatocytes are the main parenchymal cell in 17 PCW liver. We observed three distinct hepatocyte clusters (cluster 1, cluster 3, and cluster 4) distributed along with the spatial depth of 17 PCW liver (Figures 4A,B). We found that the MEG3, ITIH3, and HMGCS1 showed cluster 1-high expression. The genes FGB, IGFBP1, and A2M were more highly expressed in cluster 3, indicating that regional DEGs may be involved in forming region-specific functions of the corresponding regions. Also, hepatocyte cells in cluster 4 highly expressed several genes, including MT-CO1, MT-CO2, MT-CO3, which involved in mitochondrial electron transport and oxidative phosphorylation (Supplementary Figure 5). It was noteworthy that cluster 1 and cluster 3 mainly gathered together in the central region of the liver tissue, and cluster 4 mainly gathered together in the periphery of liver (Figure 4A). Heatmaps from differential expression analysis showed the gradually down-regulated or up-regulated genes during hepatocyte differentiation (Supplementary Figure 6A). GO analysis revealed that gradually up-regulated genes among Cluster 1-3-4 were mainly associated with regulation of planar cell polarity pathway, Wnt signaling pathway, and activation of GTPase activity, while gradually down-regulated genes were involved in the regulation of necrotic cell death, glycogen biosynthetic process, and liver regeneration. In addition, we described the cellular communication between the Cluster 1-3-4 cells (Figure 4C), providing potential signaling transduction messages for future investigation. Remarkably, Cluster 3 had more significant signaling transduction with Cluster 4 cells than Cluster 1 cells, although Cluster 3 and Cluster 1 were closer together. In addition, we identified some shared and active ligand-receptor pairs such as DLK1-NOTCH2, EFNA1-EPHA2, AGT-AGTR1, which was found both in the cellular communication of Cluster 1 cells to Cluster 4 cells and Cluster 3 cells to Cluster 4 cells.

In the scRNA-seq data of 17 PCW liver, hepatocyte cells could be further subdivided into three clusters. The DEGs among the clusters were identified. Heatmap showed the top ten marker genes for each cluster (Figure 4D). Recently, Prior et al. (2019) identified LGR5^+^ stem and progenitor cells from the hepatoblast pool at the early stage of liver development. We examined the expression patterns of LGR5 and found a fraction of LGR5^+^ hepatoblasts based on our scRNA-seq data (Figure 4E). At 8 PCW liver, 5.68% of hepatoblasts express LGR5, but the percentage of LGR5^+^ cells dropped to 3.80% at 17 PCW liver. Moreover, the existence of a subgroup of HNF4A^+^ID3^+^ hepatoblasts at 8 and 17 PCW liver (Figure 4F). We designated these HNF4A^+^ID3^+^ cells as ID3^+^ hepatoblasts. Our scRNA-seq data revealed that 25.26% (120 cells of 475 cells) of 8 PCW hepatoblasts and 15.76% (29 cells of 184 cells) of 17 PCW hepatoblasts belonged to this small cluster. In addition, DLK1 was highly expressed in ID3^+^ hepatoblasts, however, NCAM1 was not expressed in ID3^+^ hepatoblasts. Moreover, we identified two major cell states (cluster 7 and cluster 10) of megakaryocyte in the 17 PCW fetal liver (Figure 2E). The results showed that PF4, PPBP, ITGA2B, GP9, and MYL9 were up-regulated in Cluster 7, JCHAIN, IGLC3, IGLC2, IGHM, and IGKC were up-regulated in Cluster 10 (Supplementary Figure 6B).

### Erythroid Lineage Differentiation and Maturation Pathway in the Fetal Liver

**FIGURE 5:**
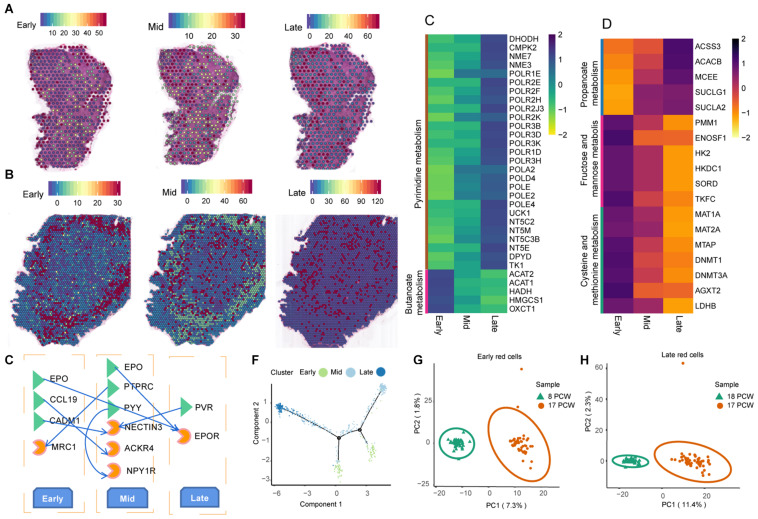
Spatially resolved erythroid lineage differentiation and maturation pathway. **(A,B)** Visualization of the distribution of red blood cells across tissue sections from the 8 PCW **(A)** and 17 PCW liver **(B)**. **(C,D)** Heatmap of gradually down-regulated or up-regulated genes during erythrocyte differentiation and their corresponding enriched KEGG pathway in 8 PCW **(C)** and 17 PCW liver **(D)**. **(E)** Cellular communication between Early-Mid-Late erythroblasts in the 17 PCW liver. **(F)** Pseudotime analysis shows the differentiation and relation of early, mid, and late erythroblasts. **(G,H)** PCA plot showing different gene expression patterns between the two embryonic stages. The PCA plot analyses the 50 spots represented in early red cells and late red cells from 8 and 17 PCW liver. PCW, post-conception weeks.

In 8 PCW liver, small clusters of red blood cells were scattered throughout the liver (Figure 5A). In 17 PCW liver, the livers were filled with red blood cells and were reddened entirely (Figure 5B). It is noteworthy that early erythroblasts were in the periphery of the liver tissue, and mid erythroblasts and late erythroblasts were scattered in the central region of the liver tissue (Figure 5B). This dramatic morphological change indicated that both human fetal livers experienced blood rapid growth and immigration during the period studied in our experiments. There were early, mid, late erythroblasts both in 8 and 17 PCW liver. To investigate the developmental process of erythrocyte, we investigated genes differentially expressed among these three types of red cells. In the 8 PCW liver, 818 genes were gradually down-regulated, and 2648 genes were gradually up-regulated during Early-Mid-Late differentiation. GO characteristics and continued maturation pathways related to human erythroid development were identified (Supplementary Figures 7, 8). The gradually up-regulated genes were enriched for glyoxylate and dicarboxylate metabolism, pyrimidine metabolism, fructose and mannose metabolism, pyruvate metabolism, and purine metabolism, while the gradually down-regulated genes were associated with butanoate metabolism (Figure 5C). In the 17 PCW liver, there were 812 genes gradually down-regulated, and 880 genes gradually up-regulated during Early-Mid-Late differentiation. GO classification and enrichment analysis was performed (Supplementary Figures 9, 10). KEGG enrichment analysis of the down-regulated genes revealed that the most significant KEGG terms include fructose and mannose metabolism, cysteine and methionine metabolism, taurine and hypotaurine metabolism, histidine and thiamine metabolism-related signaling pathway. The highly enriched terms for the up-regulated genes were involved in propanoate metabolism (Figure 5D). We performed ligand-receptor interaction analysis between Early-Mid-Late cells, and found that erythropoietin (EPO), Protein tyrosine phosphatase receptor C (PTPRC), Nectin Cell Adhesion Molecule 3 (NECTIN3), et, was discovered in the Early-Mid-Late cells communication (Figure 5E). In addition, the differentiation trajectory analysis of erythrocyte was performed and yielded one tightly connected differentiation trajectory that differentiates into five main branches (Figure 5F). This suggests that the system is maintained through one continuous rather than several disconnected lineages. Moreover, we identified 617, 674, and 503 DEGs in early, mid, and late erythroblasts between 8 PCW liver and 17 PCW liver, respectively. To obtain further insight into the differences of erythrocyte between 8 PCW liver and 17 PCW liver, we performed PCA of gene expression patterns between the two embryonic stages. We sampled 50 spots from each cell type to generate balanced datasets, and found that all the spots could be distinguished between 8 PCW and 17 PCW liver based on their transcriptional similarity (Figures 5G,H).

### Expression Patterns of the Liver Disease-Related Gene Across Fetal Liver Tissue

**FIGURE 6:**
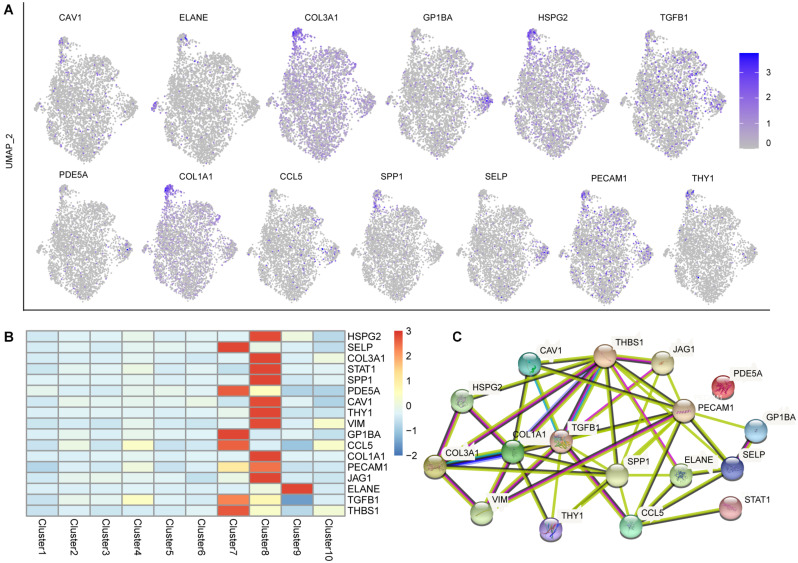
Expression patterns of the liver disease-related gene across tissue sections from the 17 PCW liver. **(A)** The UMAP plots showing the expression level of liver disease-related genes. The color changed from gray to blue as the gene expression levels increase. **(B)** Seventeen genes published and linked to liver disease are shown by a heat map based on their average gene expression level in each cell cluster. **(C)** Protein–protein interaction networks analysis of the 17 liver diseases-related genes displayed based on the degree of complexity of the nodes.

According to “the fetal and infant origins of adult disease” hypothesis (Barker, 1990), the fetal liver is subject to environmental influences that, through epigenetic mechanisms, can have sustained effects on function and, by extension, contribute to the developmental origin of adult metabolic disease (Gruppuso and Sanders, 2016). To identify genes functionally associated with the fetal origins of liver disease, we integrated our fetal liver ST data with a publicly available web resource^2^ (Pinero et al., 2020) to visually explore the cell types and spatial regions related to liver’s disease. 17 genes published and linked to liver disease mainly expressed in megakaryocyte and endothelial, hardly expressed in any other cell types (Figures 6A,B). For example, SPP1, TGFB1, JAG1, STAT1, and VIM were linked to congenital biliary atresia, and were confirmed to be strongly expressed in the endothelial and megakaryocyte. The expression of TGFB1 and THBS1 (Congenital hepatic fibrosis-related) were also highly detected in megakaryocyte and endothelial. In addition, childhood Hepatocellular Carcinoma related genes SPP1 and TGFB1 were also strongly expressed in the endothelial and megakaryocyte. It was worth noting that SPP1 and TGFB1 genes are not only associated with congenital (and childhood) liver disease, but also with adult liver disease. Here, we also analyzed the specific expression of some genes which related to adult liver disease. For example, hepatitis B-related genes (GP1BA, HSPG2, CCL5, and SPP1), fatty liver diseases-related genes (COL3A1, CCL5, and SPP1), alcoholic liver diseases-related genes (CAV1, ELANE, CCL5, and SPP1), primary biliary cirrhosis-related genes (TGFB1, PDE5A, COL1A1, CCL5, and SPP1), and primary malignant liver neoplasm-related genes (SELP, PECAM1, HSPG2, and THY1), mainly expressed in endothelial and megakaryocyte, hardly expressed in any other cell types.

In order to further reveal this phenomenon, we analyzed the expression level of these 17 liver diseases-related genes in 17 PCW embryonic kidney^3^. Except PECAM1, the rest of the genes expressed in 17 PCW kidney (Supplementary Figure 11). But the expression pattern was different from that in liver, not mainly expressed in megakaryocyte and endothelial, which indicated that these liver disease-related genes have a unique gene expression patterns in the embryonic liver. Besides, the protein interaction network analysis of these 17 liver diseases-related genes revealed a key role for TGFB1, COL1A1, SPP1, PECAM1, and THBS1 in the expression network, and no interaction of PDE5A with other genes (Figure 6C).

### Weighted Gene Co-expression Network Analysis (WGCNA) of Embryonic Liver Development Genes

**FIGURE 7:**
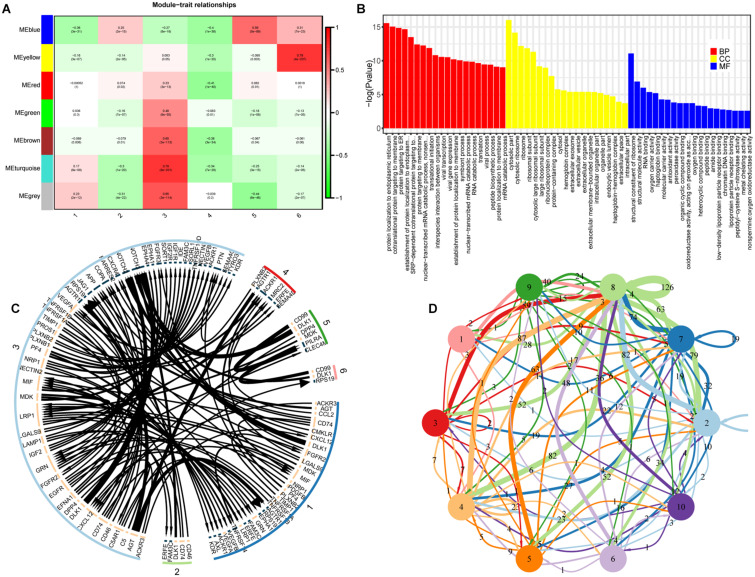
Co-expression networks analysis and cellular communication analysis of the cell clusters in embryonic liver. **(A)** The hierarchical clustering tree shows each co-expression modules in the 8 PCW liver. **(B)** The heatmap shows the Spearman correlation of co-expression modules in the 8 PCW liver, **(C,D)** Cellular communication analysis of the cell clusters in the 8 PCW liver and 17 PCW liver. PCW, post-conception weeks.

To better understand liver development relevant changes in gene regulation and interactions between cell types, we used the WGCNA algorithm to construct an unbiased co-expression analysis of our ST data based on genes differentially expressed in the major cell clusters (Zhang and Horvath, 2005). We identified five major co-expression modules in 8 PCW liver, and define two gene expression modules in 17 PCW liver (correlation index >0.56) (Figure 7A and Supplementary Figure 12A). Each module showed distinct correlation profiles across cell clusters. Examining these modules revealed that many of the modules were comprised of genes preferentially expressed in specific cell clusters. In addition, high-correlation modules tended to contain enriched KEGG pathways, GO biological process, and protein–protein interaction (PPI) network, which further highlighted the collaborative mode in the functioning of genes (Figure 7B and Supplementary Figures 12, 13B,C). In the 8 PCW liver, KEGG analysis shows that module-blue was enriched for several pathways, including ribosome, DNA replication, and glutathione metabolism (Supplementary Figure 12A). The co-expression network of the 23 genes of module-yellow revealed a vital role for PF4 and PPBP in the expression network (Supplementary Figure 13D). In addition, cellular communication and signaling transduction regulation between cell clusters (or cell type) are the earliest process in fetal liver development. To investigate cellular communication and spatial cross-talk in the fetal liver, we performed ligand-receptor interaction analysis, and found that there were active cellular communications between cell clusters (Figures 7C,D and Supplementary Figure 14A,B). The ligand-receptor pairs such as CD74-MIF, DLK1-NOTCH3, IGF2-IGF1R, AGT-AGTR1, and CCL2-ACKR1 were more frequently found in 8 PCW liver, and the ligand-receptor pairs such as ESAM-ESAM, TIMP1-FGFR2, JAG1-NOTCH3, LTBR-LTB, and PLXNB2-PTN were more frequently found in 17 PCW liver. Moreover, there were 129 ligand-receptor pairs shared by 8 PCW and 17 PCW liver (Supplementary Figure 14C).

### Cell Cycling Stage Analysis of the Fetal Liver Cells

**FIGURE 8:**
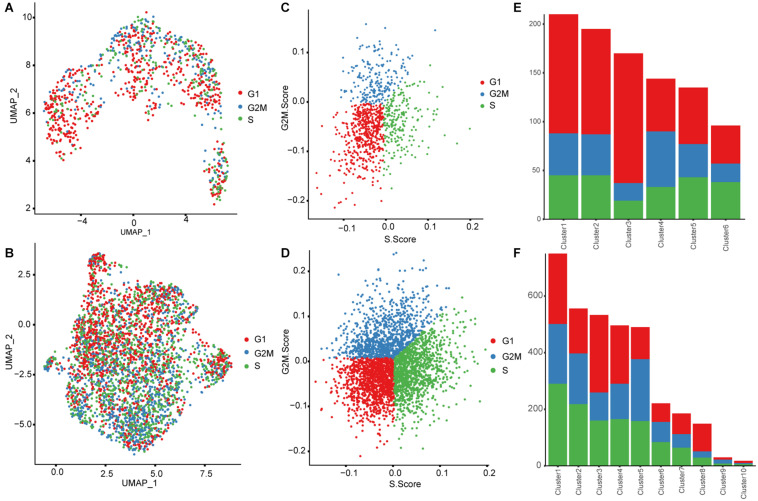
Cell cycling stage analysis of the fetal liver cells. **(A–D)** UMAP plots and Cell Cycle Scoring Plot showing the cell cycle phases of in 8 PCW **(A,C)** and 17 PCW liver cells **(B,D)**. **(E,F)** Plot showing the proportional contribution of cells in different phases (G1, G2/M, and S) identified in each cell cluster from 8 PCW **(E)** and 17 PCW liver **(F)**.

Using the CellCycleScoring algorithm, we identified the cell cycle phases of fetal liver cells (Figure 8), and found that the proportion of proliferative hepatocytes (the cells in S and G2/M phases) increased in 17 PCW liver (∼57.95%) compared with those in 8 PCW liver (∼21.76%). This phenomenon also existed in the development of red blood cells. Specifically, these proliferative cells increased from 8 PCW (Early, ∼59.38%; Mid, ∼57.04%; Late, ∼62.50%) to 17 PCW (Early, ∼70.14%; Mid, ∼73.33%; Late, ∼76.94%) in humans fetal liver tissue. Therefore, the proliferation rate of hepatocytes and erythrocyte increased throughout development in our ST data.

## Discussion

Fetal liver development is a complex process that includes immigration, differentiation, and interaction of many cell lineages derived from the mesoderm and endoderm. The development of the human fetal liver *in utero* has remained poorly understood. In this study, we applied a method for the identification and spatial mapping of distinct cell states, subpopulations, and cell types within fetal liver tissue. The method starts with the characterization of clusters and cell types present in live tissue by scRNA-seq, and, in parallel, the identification of transcriptomic regions by ST. Through MIA, we can integrate ST data and scRNA-seq data perfectly, and analyze the data from regions of interest in an unbiased way. By applying MIA, we mapped the location of distinct cell types (kupffer, hepatocyte, early erythroblast, mid erythroblast, late erythroblast, neutrophil, megakaryocyte, and endothelial) and subpopulations (2 different clusters of mid red cell in 8 PCW liver, three hepatocyte clusters and 2 Megakaryocyte clusters in 17 PCW liver) in the fetal liver tissue, and the relationships among cell types (clusters).

We identified three hepatocyte sub-clusters both in ST and scRNA-seq data, which showed different gene expression and functional enrichment in each sub-clusters. It suggested that hepatocytes in different regions may perform different functions. Several active ligand-receptor pairs, such as DLK1-NOTCH2, EFNA1-EPHA2, AGT-AGTR1, have been identified in the cellular communication of these sub-clusters. The liver is the principal hematopoietic organ in the human fetus from the 6th week through mid-gestation (Suskind and Muench, 2004). We identified the differentiation and development of erythrocytes in fetal liver tissue as early as 8 PCW. The results showed that human fetal livers experienced blood rapid growth and immigration during the period studied in our experiments. A large number of genes were gradually up-regulated or down-regulated during Early-Mid-Late differentiation. KEGG pathways analysis indicated that metabolic changes are crucial for the maturation of human erythroblasts. In addition, we conducted an unbiased co-expression analysis on the liver spatial data, resulting in five and two expression modules in 8 and 18 PCW liver, respectively. High-correlation modules tended to contain enriched KEGG pathways, PPIs, and GO biological processes, which further highlighted the collaborative mode in the functioning of genes. Moreover, the proliferation rate of hepatocytes and erythrocyte increased throughout development in our ST data. However, Wang and co-workers found the opposite results. They found that, in both humans and mice, the proliferation rate of hepatoblasts/hepatocytes decreased throughout development (Wang et al., 2020). These differences between studies may be related to both the selection of embryonic stage and the methods.

Since Barker’s publication of “The fetal and infant origins of adult disease” in Barker (1990), significant emphasis has been placed on the intrauterine environment and its effect on adult disease. Environmental changes will cause the body’s response and adjustment. In the embryonic stage, all tissues and organs are in the most sensitive stage of genesis, differentiation and development. If the intrauterine environment is not conducive to embryo differentiation and development, then the embryo will make structural and functional changes, including epigenetic mechanisms, this adaptive change of embryo will cause serious or even irreversible damage to its life (Carpinello et al., 2018; Simeoni et al., 2018). The specific extent of damage depends on the type, nature, degree, timing and duration of intrauterine environmental changes, and is also related to the genetic susceptibility of the fetus. Relevant to this investigation, Hochane et al. (2019) analyzed the kidney disease-related genes in human fetal kidney based on single-cell transcriptomic. In this study, we focused on the expression of liver disease-related genes in embryonic liver, and found that 17 genes published and linked to liver disease mainly expressed in megakaryocyte and endothelial, hardly expressed in any other cell types. Transforming growth factor-b1 (TGF-b1) signaling in hepatic stellate cells (HSCs) plays a crucial role in liver fibrosis by initiating profibrotic signaling in HSCs and collagen synthesis (Ghafoory et al., 2018), but the source of TGF-b1 is unclear. Here we show that megakaryocytes are rich in TGF-b1. Further studies are required to evaluate whether liver fibrosis can be prevented by blocking megakaryocyte and TGF-b1 activation during acute liver injury. In addition, liver sinusoidal endothelial cells (LSECs) form the wall of the hepatic sinusoids, which have unique morphology and function (Ni et al., 2017). These cells contain many fenestrae with uniform diameters of 100–150 nm. Hepatotropic viruses (hepatitis B virus and hepatitis C virus, etc.) usually pass through the protective filter constructed by LSECs to gain access to the liver parenchyma. LSECs constitutively express large amounts of anti-inflammatory cytokines (e.g., TGF-β), co-stimulatory molecules, and major-histocompatibility complex I-restricted antigens, which shift the hepatic immune balance toward tolerance (Limmer et al., 2005). Because many patients with chronic hepatitis often have liver fibrosis (Yamazaki et al., 2017), it is vital to know the change and function of LSECs during the development of liver diseases. There are good prospects for clinical diagnosis and new targeted therapy in the area of LSECs for liver diseases.

## Conclusion

In summary, the combination of scRNA-seq data with spatial information allowed us to identify a comprehensive set of gene probes that could summarize the spatial and cellular information and provide a clear and comprehensive understanding of all cell types’ differentiation processes in the human fetal livers. The results obtained from this study may facilitate the future study of pathogenic mechanisms and the identification of therapeutic targets in immune and infectious liver diseases.

## Data Availability Statement

Raw data files of the Spatial Transcriptomics Sequencing have been deposited in NCBI’s Gene Expression Omnibus under the GEO Series accession number GSE167096.

## Ethics Statement

This study was conducted following the tenets of the Declaration of Helsinki and was approved by the Ethics Committee of the Shenzhen People’s Hospital, China (ref. no. LL-KY-2019591). The patients/participants provided their written informed consent to participate in this study.

## Author Contributions

YD, MO, and DT: conceptualization. XH and YY: methodology and investigation. XH, PL, ZZ, WH, RZ, and XL: writing, editing, and reviewing. YD, XH, and DT: funding acquisition and supervision. All authors contributed to the article and approved the submitted version.

## Conflict of Interest

The authors declare that the research was conducted in the absence of any commercial or financial relationships that could be construed as a potential conflict of interest.

## Footnotes

1 https://satijalab.org/seurat/install.html

2 https://www.disgenet.org/search

3 http://humphreyslab.com/SingleCell/
