# Supplementary material for: Integrating Spatial Transcriptomics and Single-Cell RNA-seq Reveals the Gene Expression Profling of the Human Embryonic Liver
Source: Front Cell Dev Biol. 2021 May 20;9:652408. doi: 10.3389/fcell.2021.652408 (PMC8173368; doi:10.3389/fcell.2021.652408)
Supplement: Supplementary file 1 [file Data_Sheet_1.doc]

**Supplementary Figures**


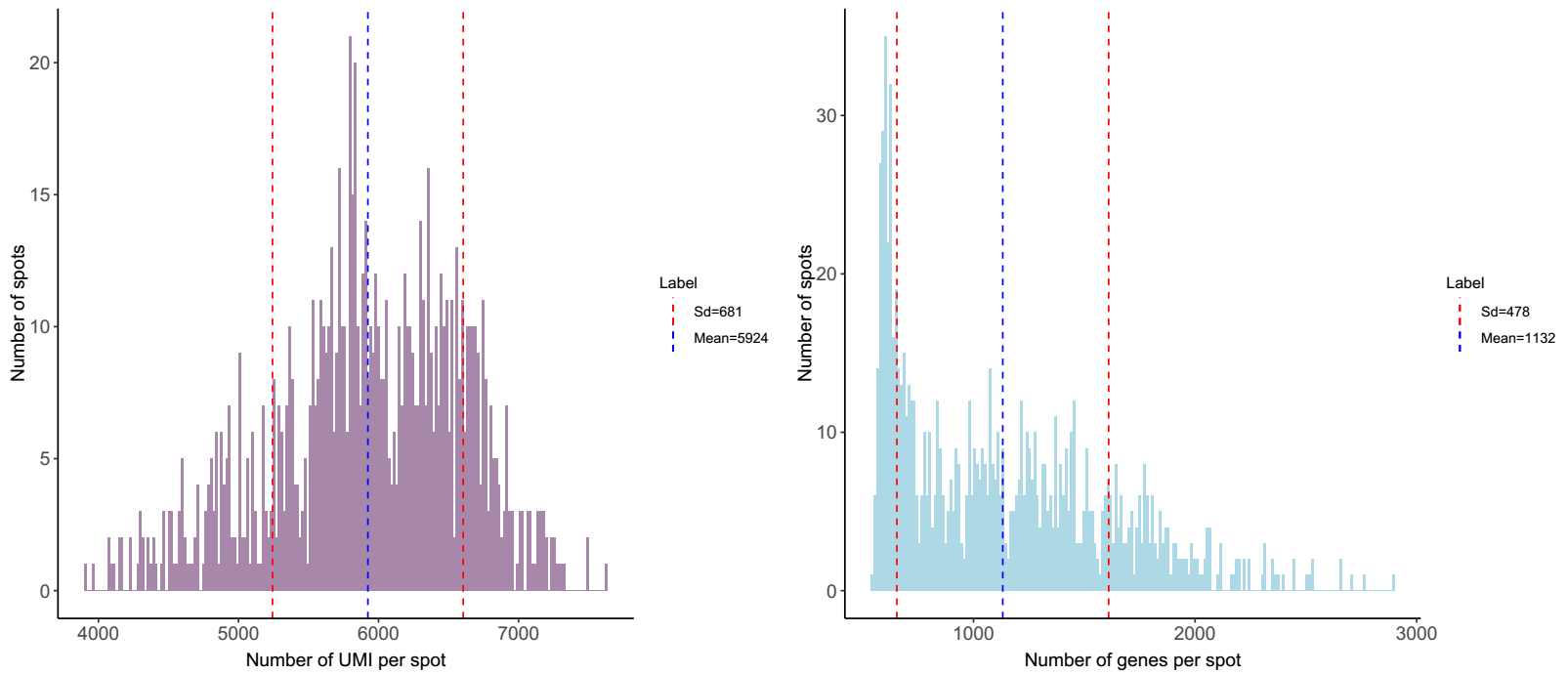


A

B


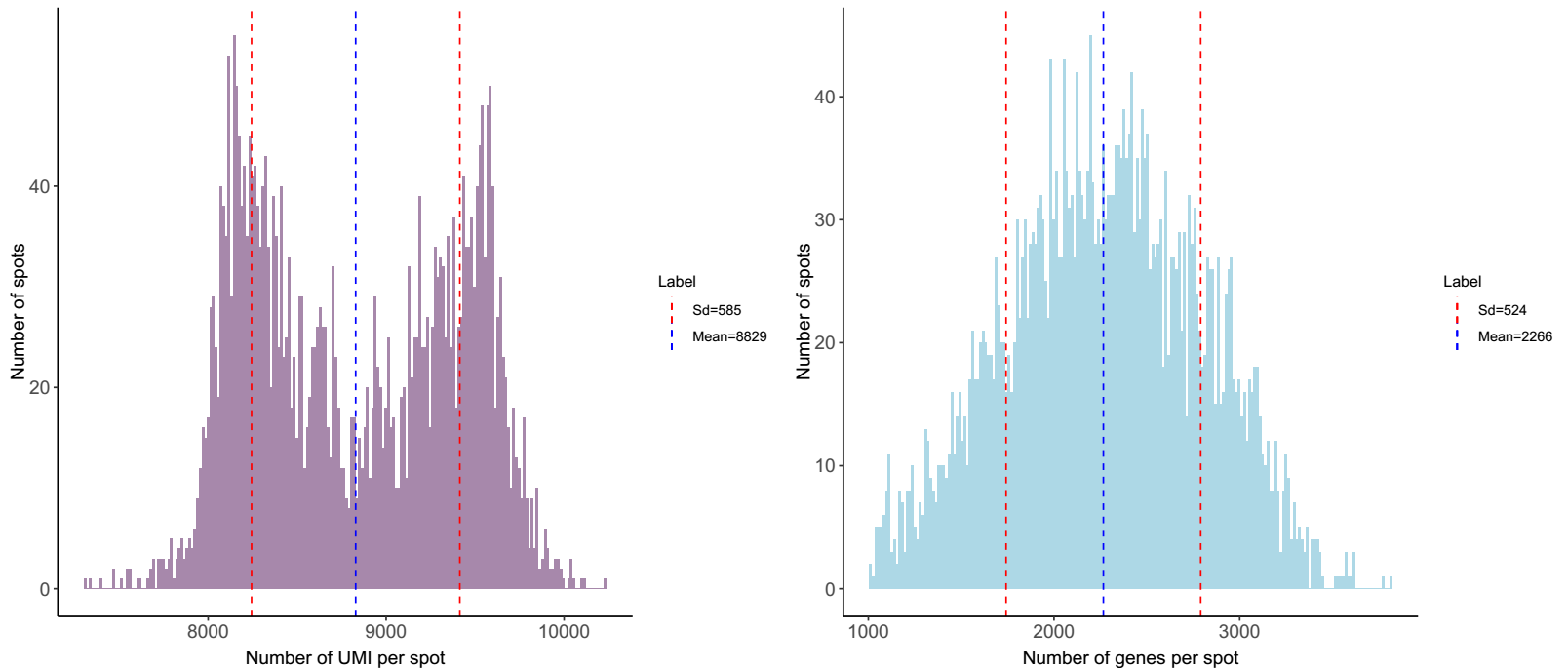


C

D

**Figure S1**. (A, B) Distribution of the number of transcripts (A) and genes (B) detected per spots in the 8 PCW liver. (C, D) Distribution of the number of transcripts (D) and genes (C) detected per spots in the 8 PCW liver. Blue dashed lines indicate mean values while the red dashed lines indicate the standard deviation.


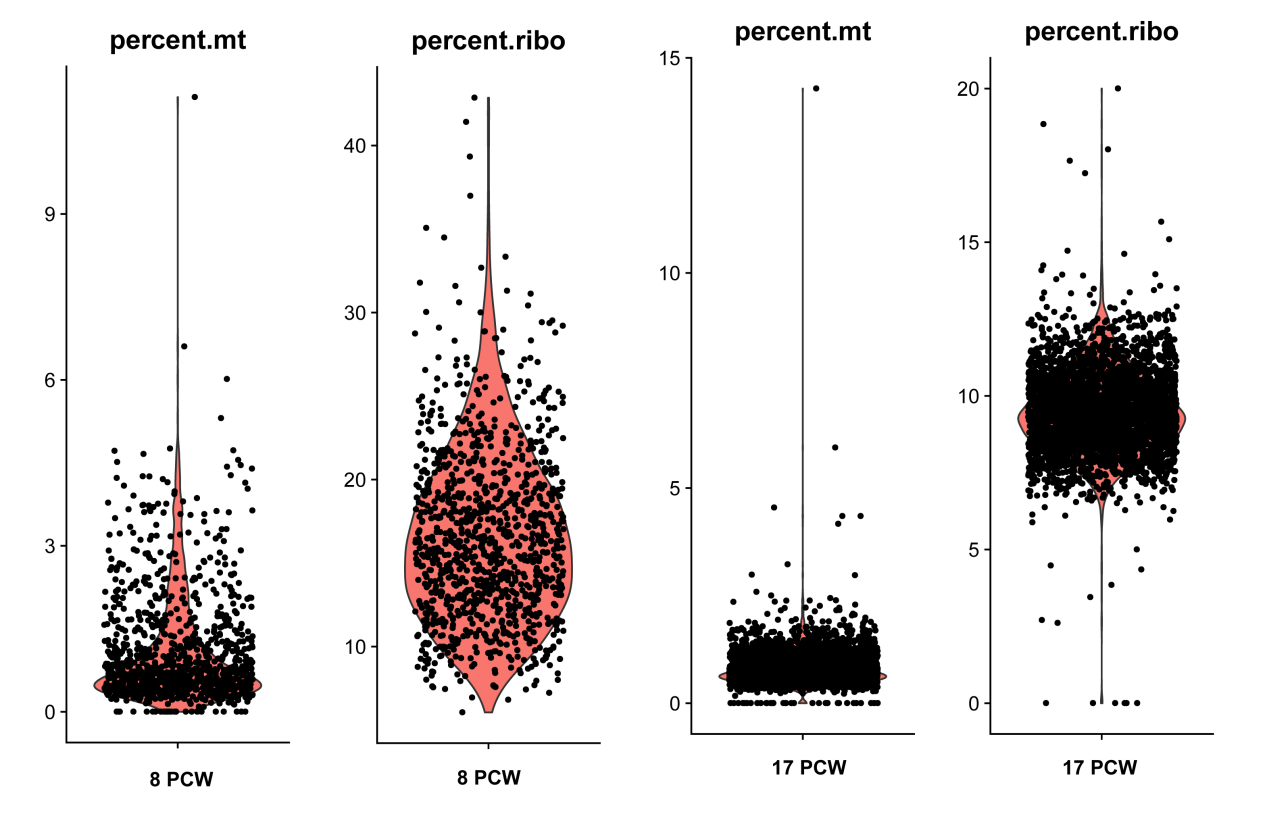


**Figure S2**. Quality Control. The percentage of UMIs that derived from mitochondrial and ribosomal genome detected per spots for 8 PCW liver and 17 PCW liver.


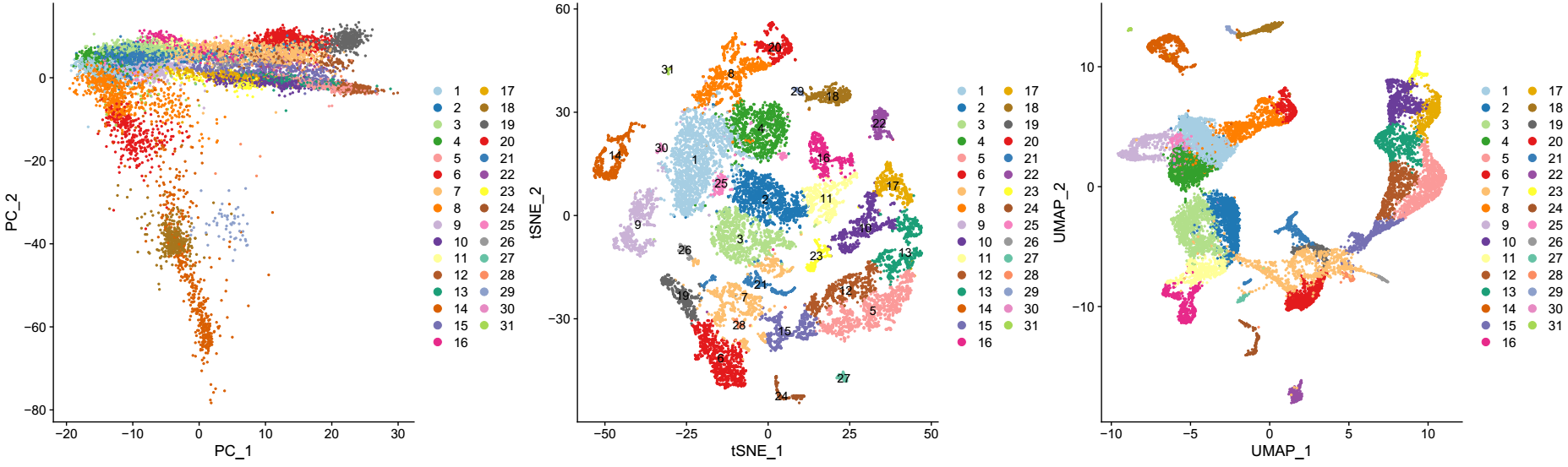


A

B


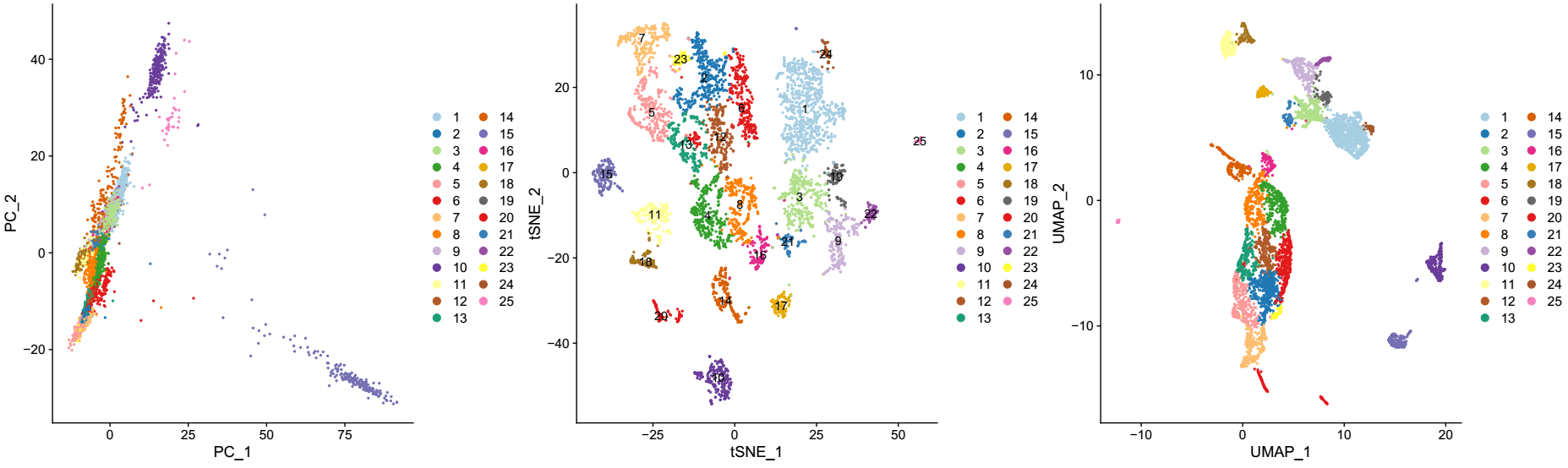


C

D

**Figure S3**. Dimensionality reduction and clustering of the scRNA-seq data of 8 PCW liver based on PCA (A) and t-SNE (B). Dimensionality reduction and clustering of the scRNA-seq data of 17 PCW liver based on PCA (C) and t-SNE (D).


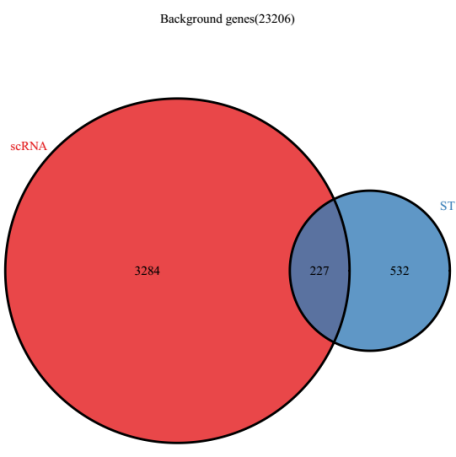

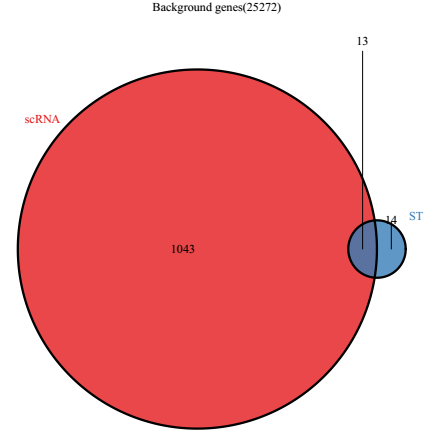


AA

BA

**Figure S4**. Venn diagram showing the overlaps of specifically expressed genes of Kupffer cell and Megakaryocyte between ST and scRNA-seq data.


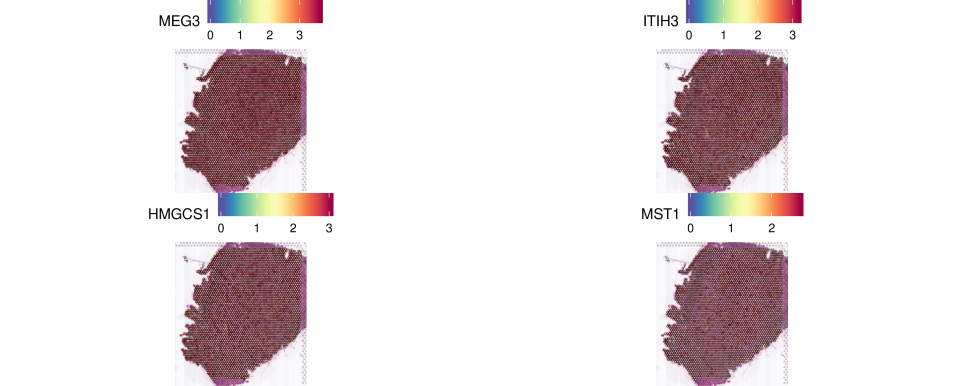


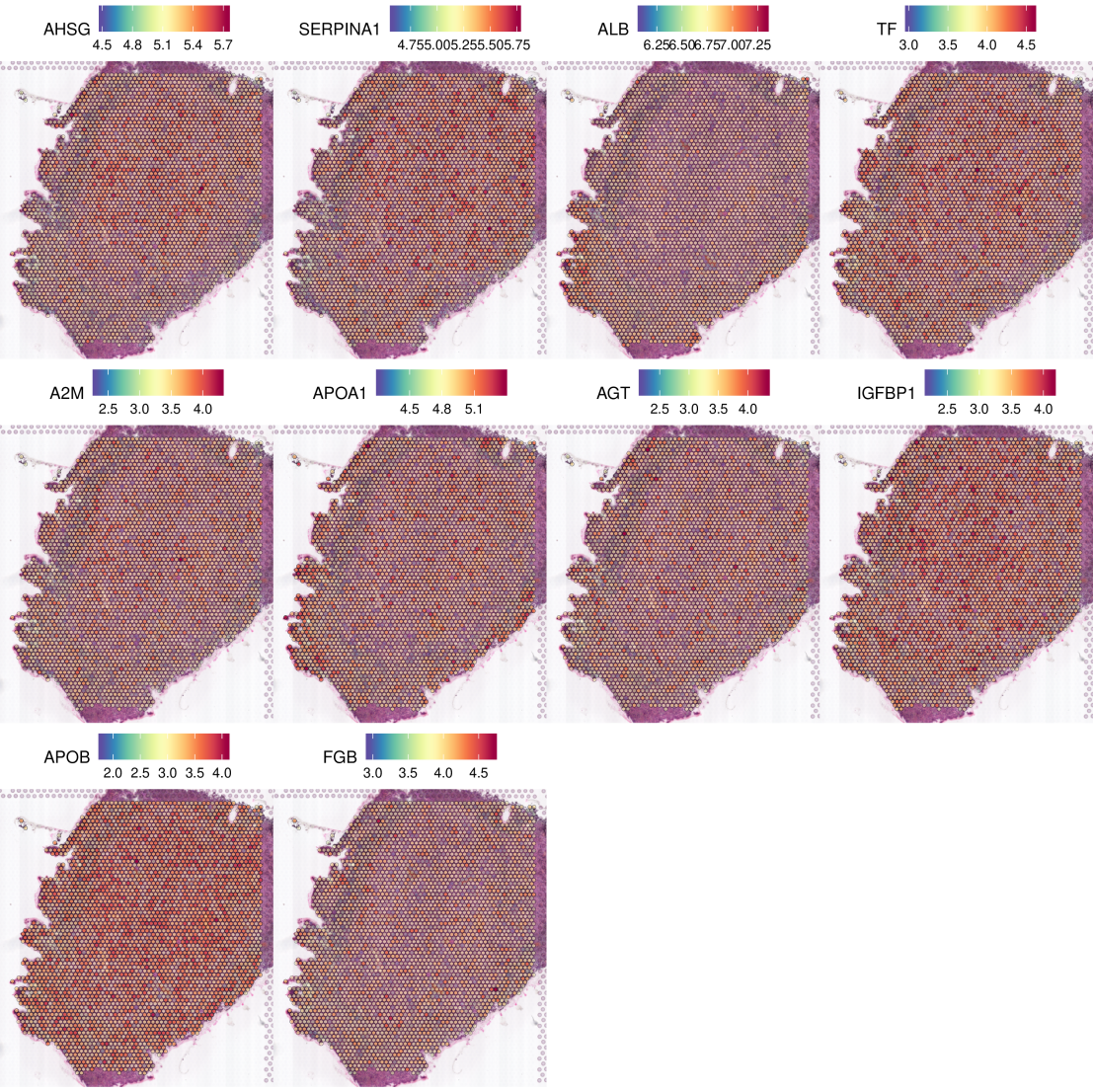


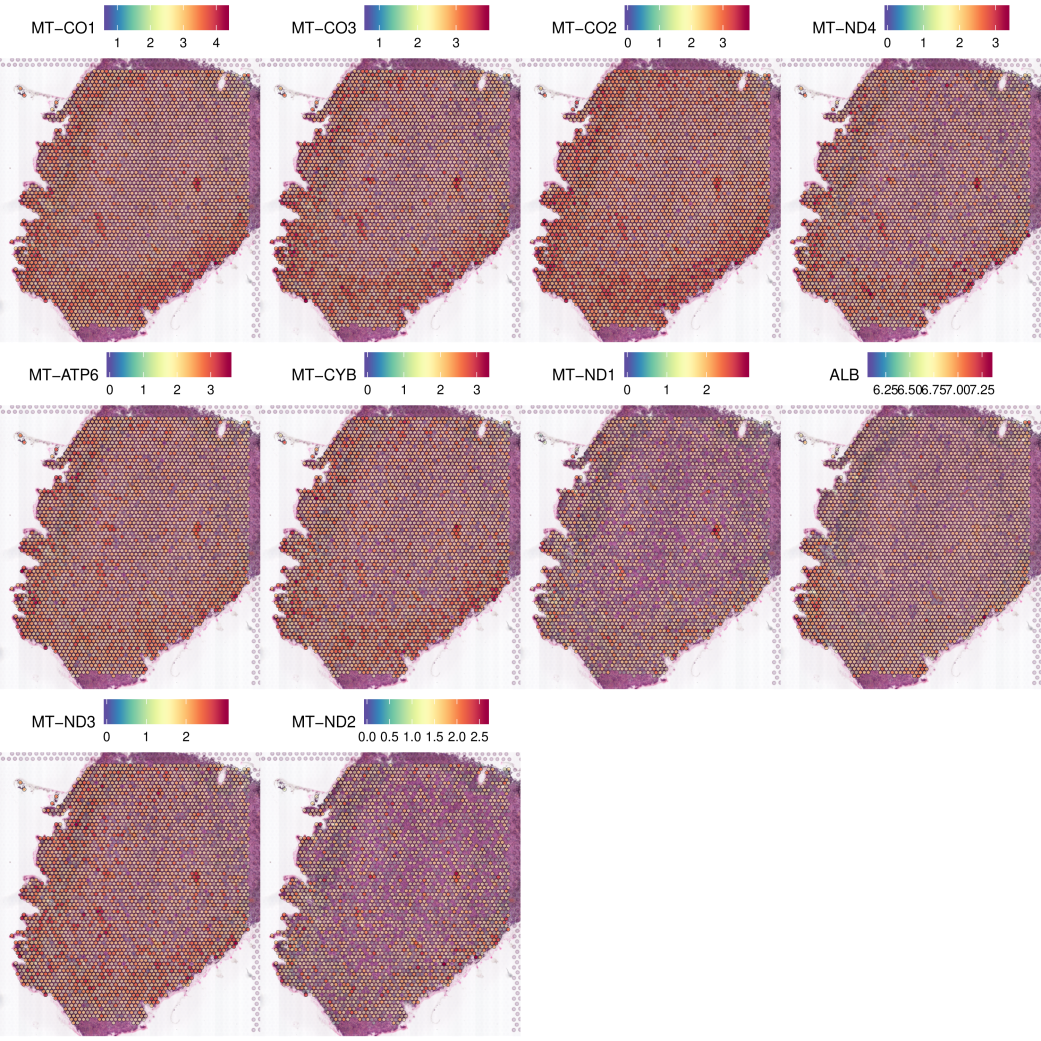


**Figure S5**. Spatially resolved heatmaps across tissue sections from the 17 PCW liver, showing spatial gene expression patterns of MEG3, HMGCS1, AHSG, SERPINA1, MT-CO1, and MT-CO3. The color changed from grey to red as the gene expression levels increase.


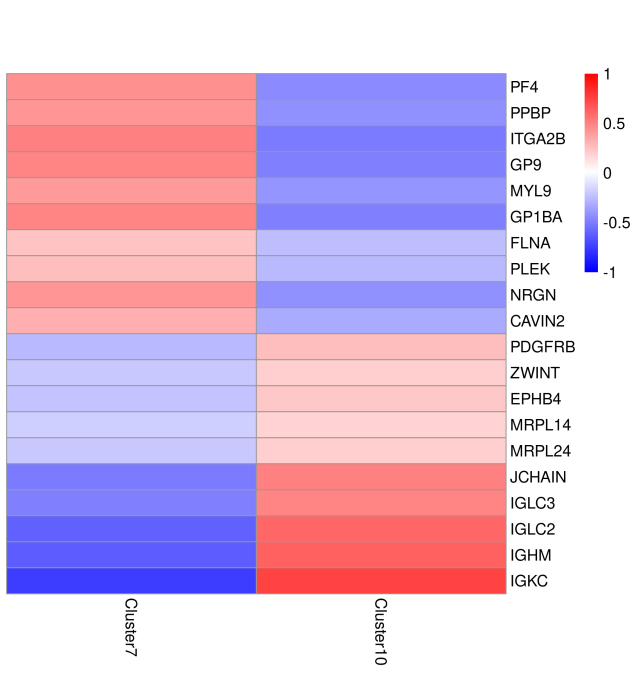

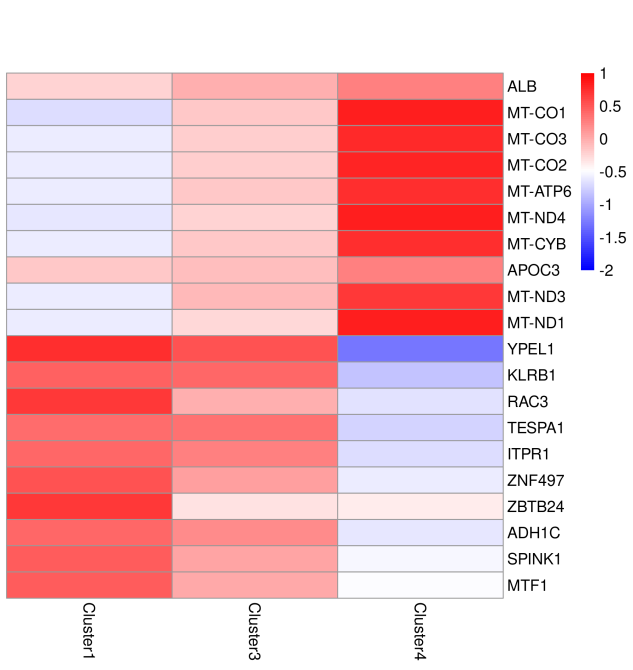


AA

BA

**Figure S6**. Gene expression profiles showed cell heterogeneity in different sub-clusters. (A) Heatmap showing the gradually down-regulated or up-regulated genes during hepatocyte differentiation. (B) Heatmap showing the expression level of the top 10 up- and down-regulated genes between the two sub-clusters of megakaryocyte.


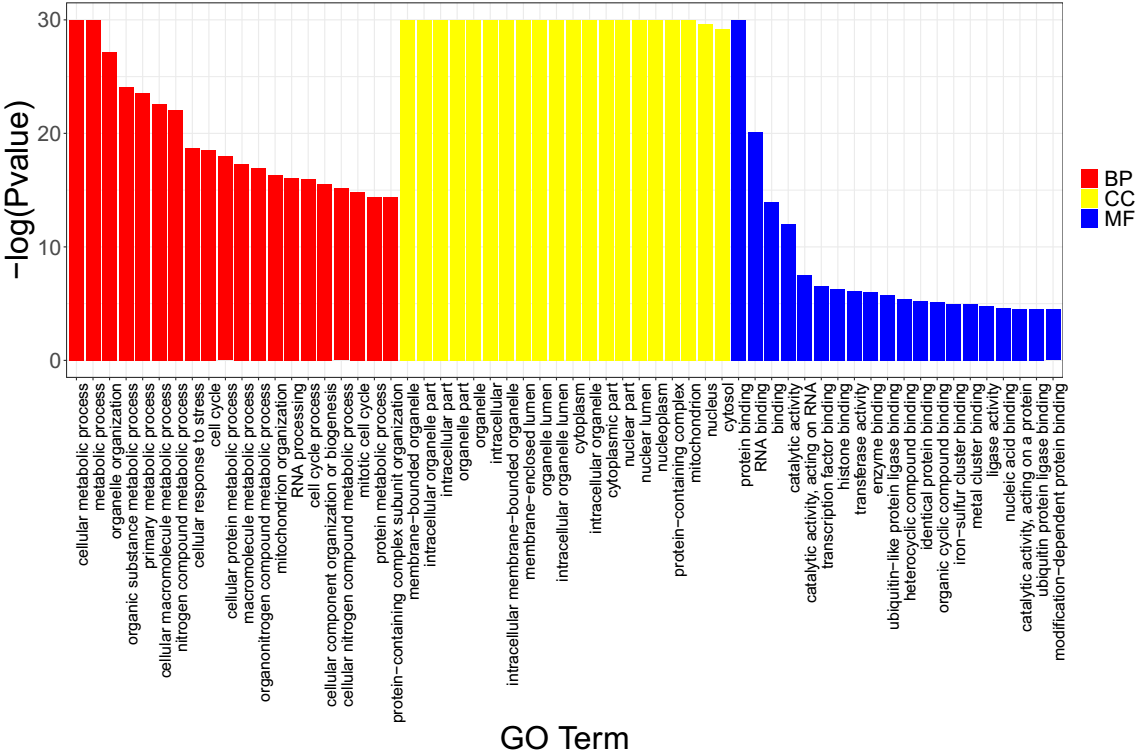


AA

BA

CA


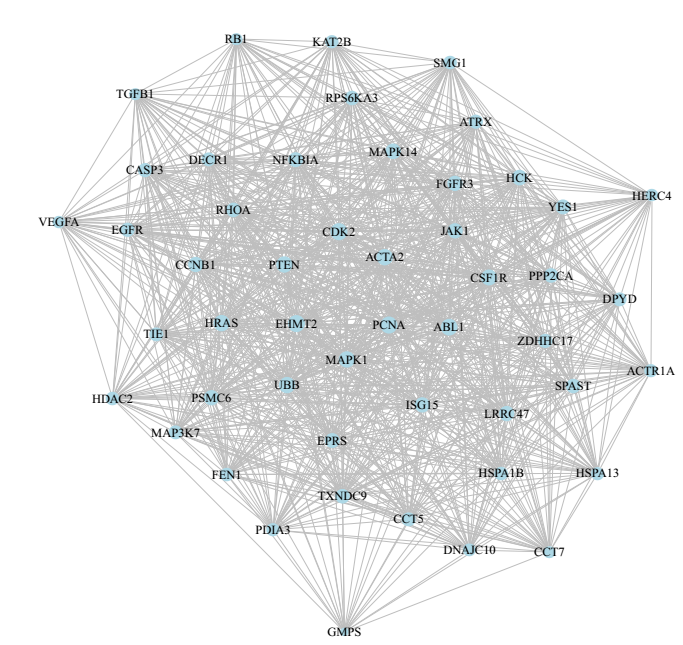

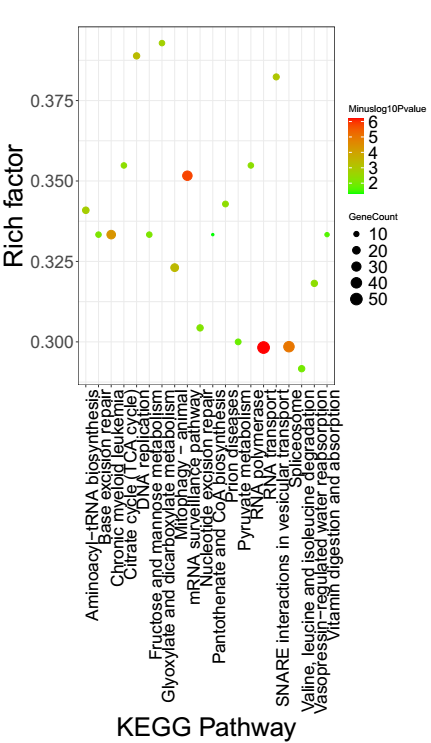


**Figure S7**. Analysis of gene expression differences during the developmental process of erythrocyte in 8 PCW liver. (A, B) Gene Ontology (GO) analysis (A) and KEGG pathway analysis (B) of gradually up-regulated genes during Early-Mid-Late differentiation. The top 20 pathways were displayed. Size of the circles depicts the gene count, and its color depicts significance levels. (C) Protein-protein interaction networks analysis of the gradually up-regulated genes during Early-Mid-Late differentiation, which were displayed base on the degree of complexity of the nodes.


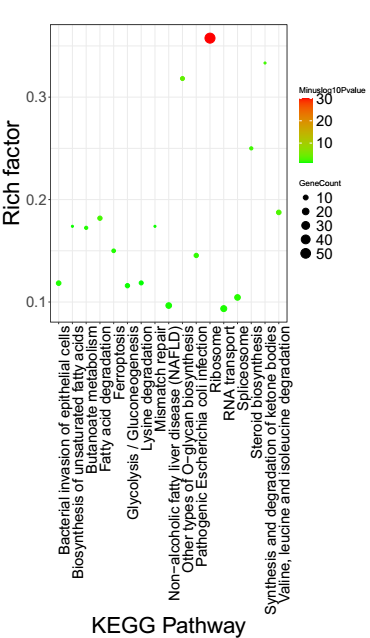

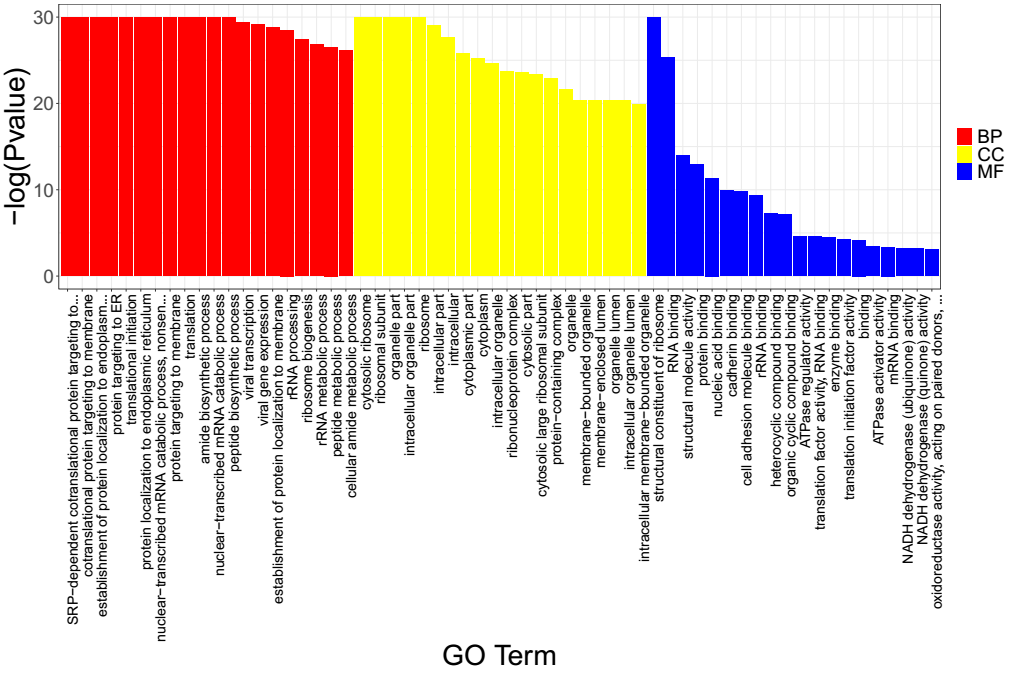


AA


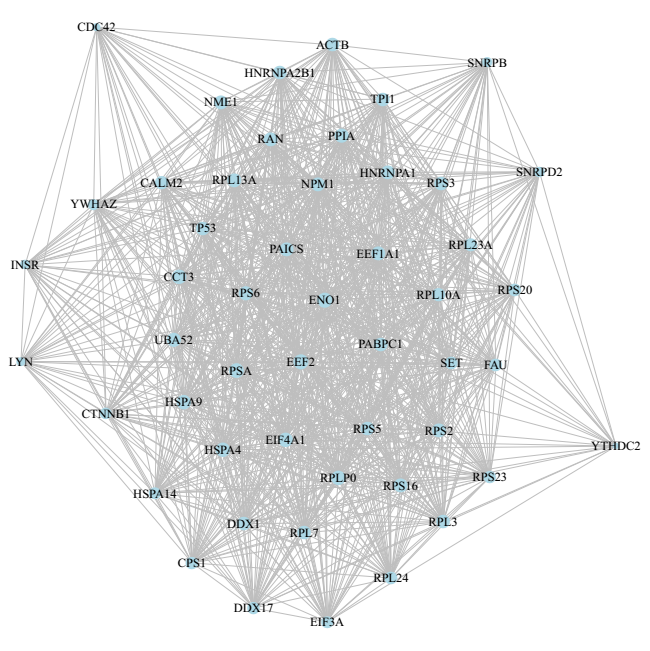


BA

CA

**Figure S8**. Analysis of gene expression differences during the developmental process of erythrocyte in 8 PCW liver. (A, B) Gene Ontology (GO) analysis ( A) and KEGG pathway analysis (B) of gradually down-regulated genes during Early-Mid-Late differentiation. The top 20 pathways were displayed. Size of the circles depicts the gene count, and its color depicts significance levels. (C) Protein-protein interaction networks analysis of the gradually down-regulated genes during Early-Mid-Late differentiation, which were displayed base on the degree of complexity of the nodes.


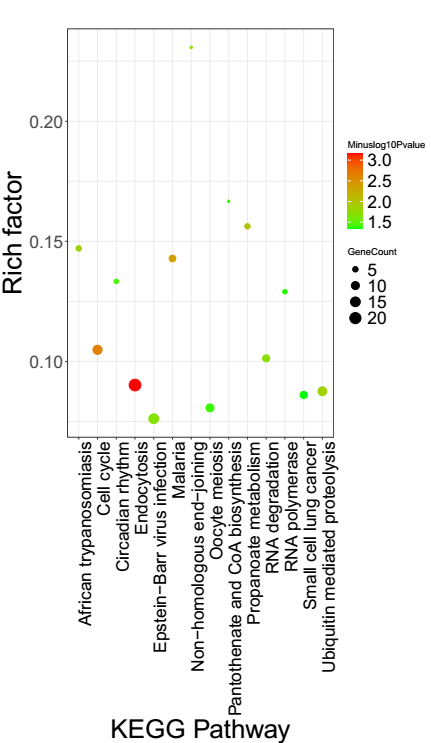

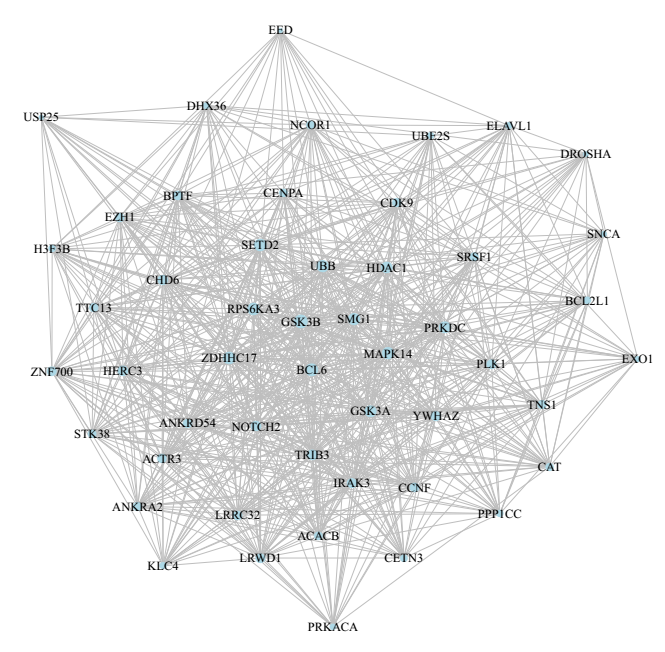

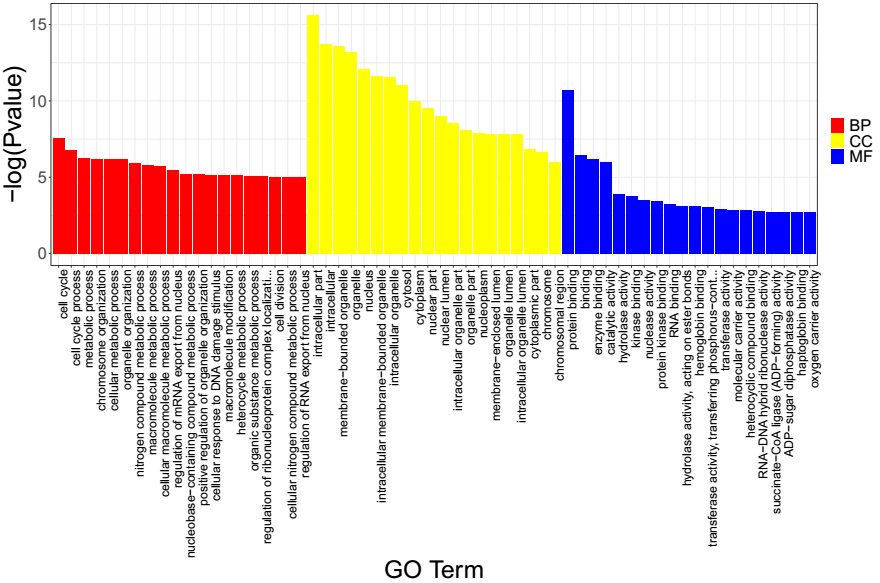


AA

BA

CA

**Figure S9**. Analysis of gene expression differences during the developmental process of erythrocyte in 17 PCW liver. (A, B) Gene Ontology (GO) analysis ( A) and KEGG pathway analysis (B) of gradually up-regulated genes during Early-Mid-Late differentiation. The top 20 pathways were displayed. Size of the circles depicts the gene count, and its color depicts significance levels. (C) Protein-protein interaction networks analysis of the gradually up-regulated genes during Early-Mid-Late differentiation, which were displayed base on the degree of complexity of the nodes.


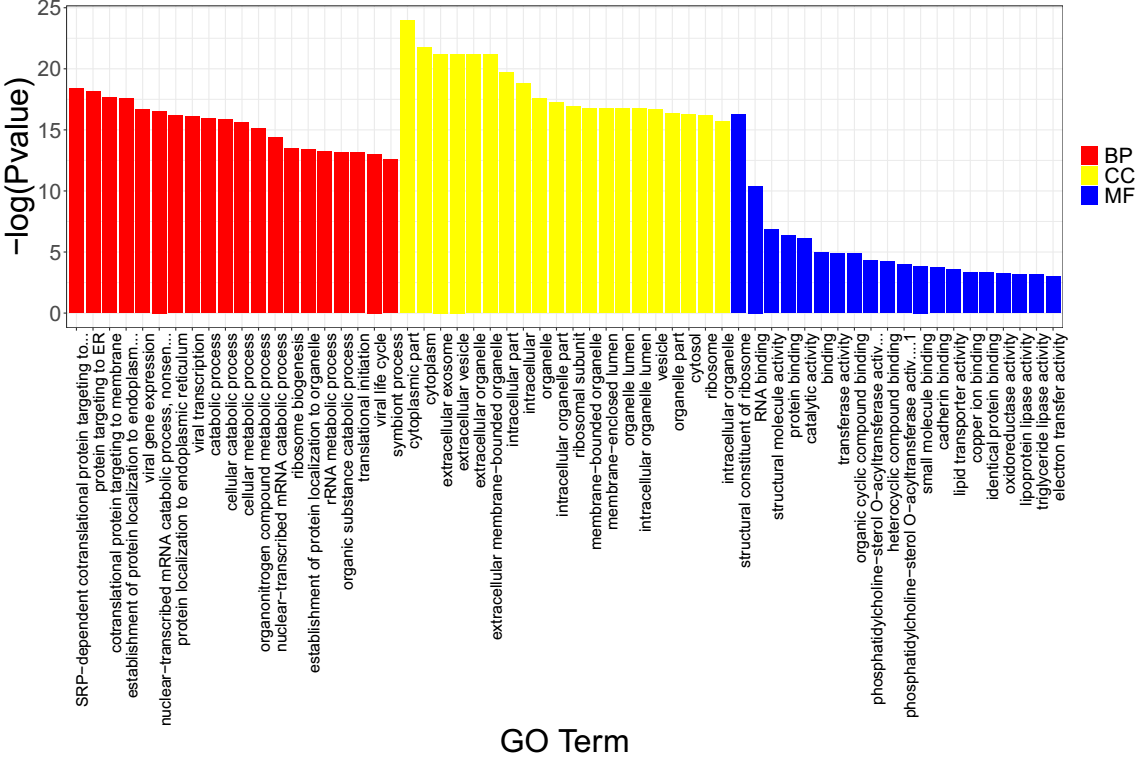

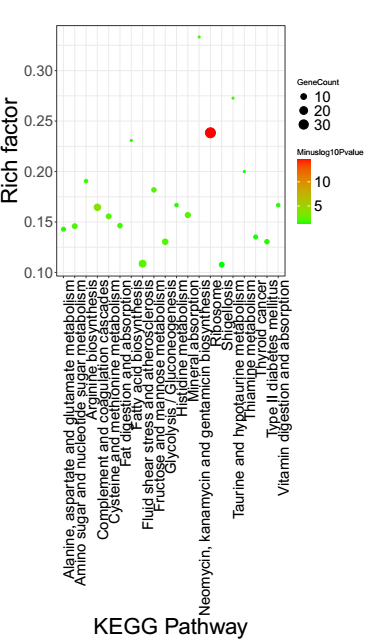


AA


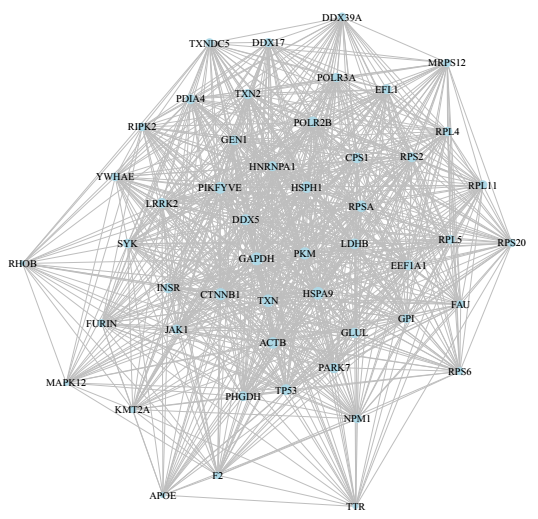


BA

CA

**Figure S10**. Analysis of gene expression differences during the developmental process of erythrocyte in 17 PCW liver. (A, B) Gene Ontology (GO) analysis ( A) and KEGG pathway analysis (B) of gradually down-regulated genes during Early-Mid-Late differentiation. The top 20 pathways were displayed. Size of the circles depicts the gene count, and its color depicts significance levels. (C) Protein-protein interaction networks analysis of the gradually down-regulated genes during Early-Mid-Late differentiation, which were displayed base on the degree of complexity of the nodes.


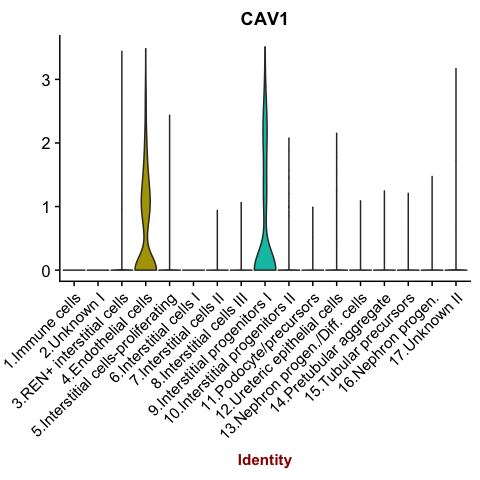

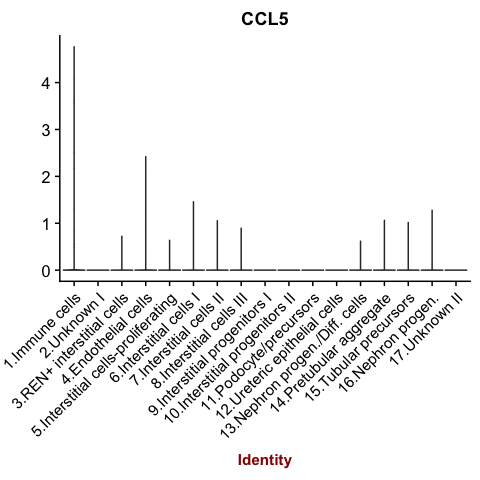


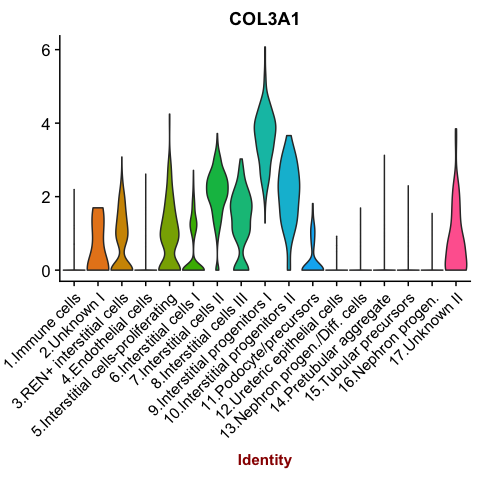

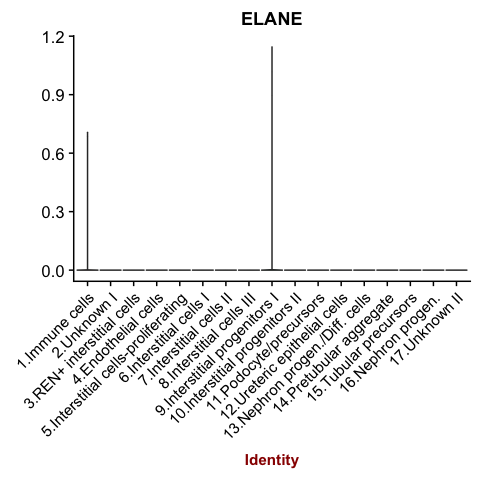


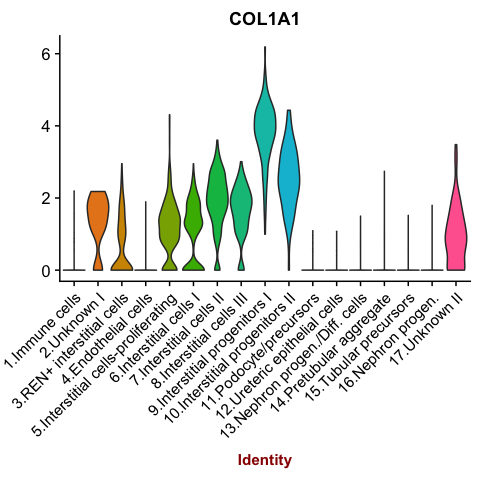

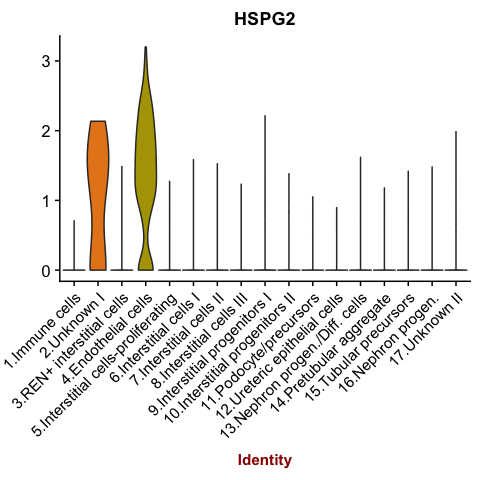


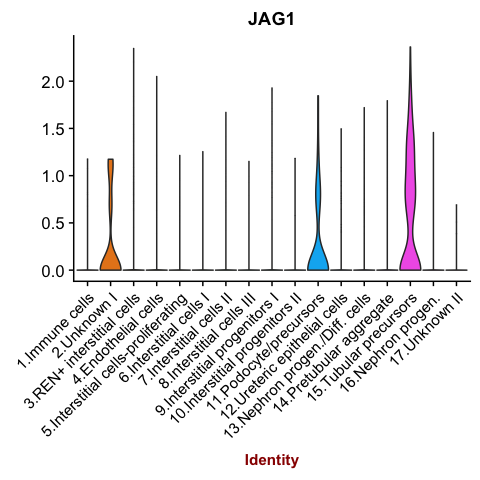

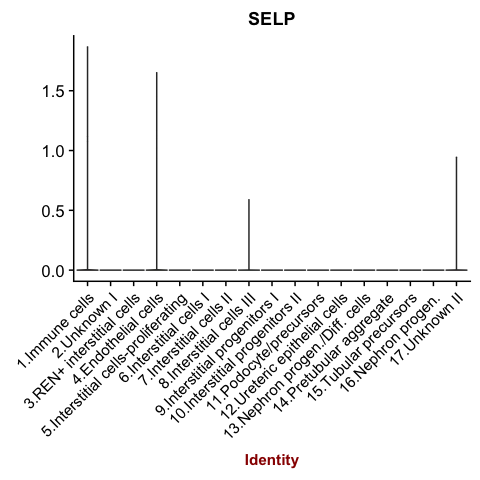


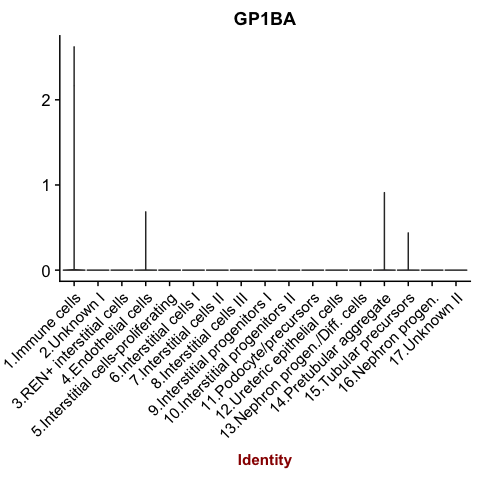

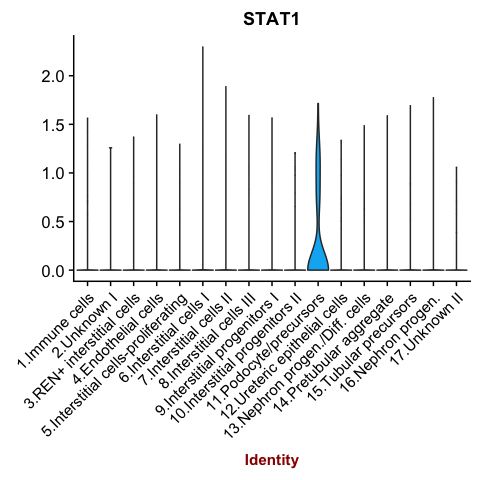


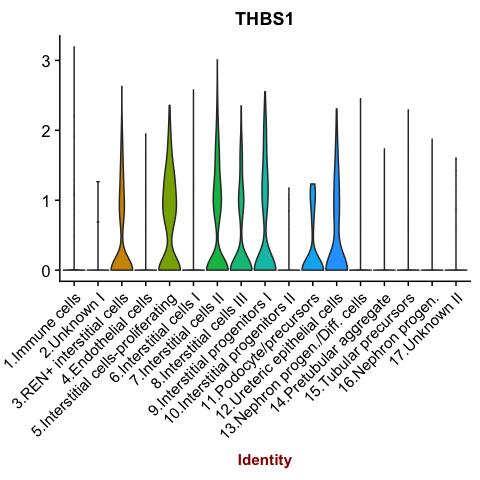

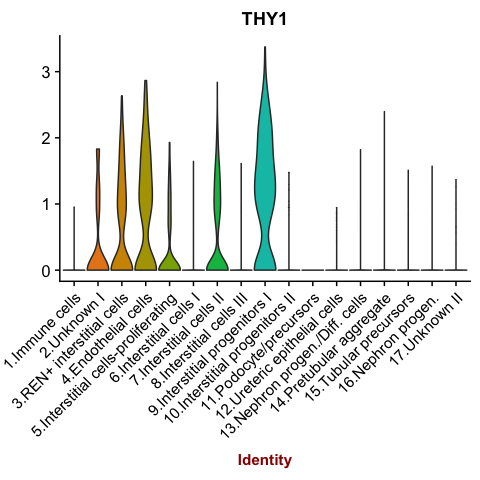


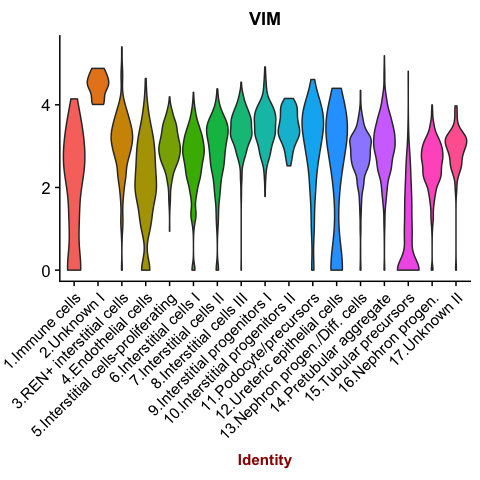

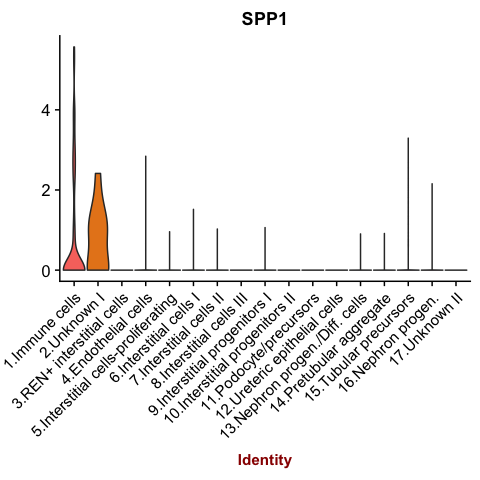


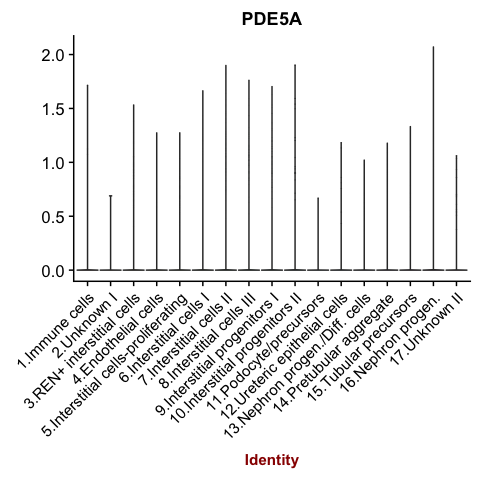

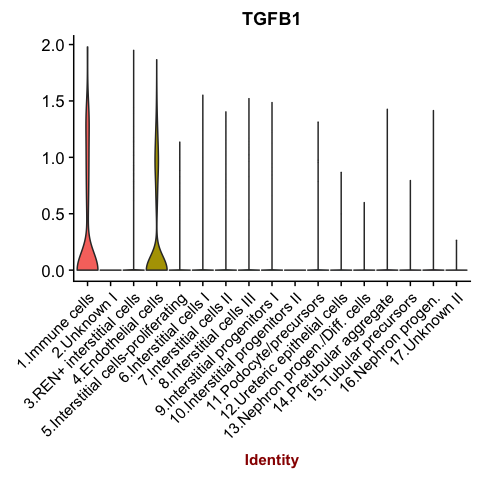


**Figure S11**. The expression pattern of the 16 liver diseases-related genes in 17 PCW embryonic kidney. PCW, postconception weeks.


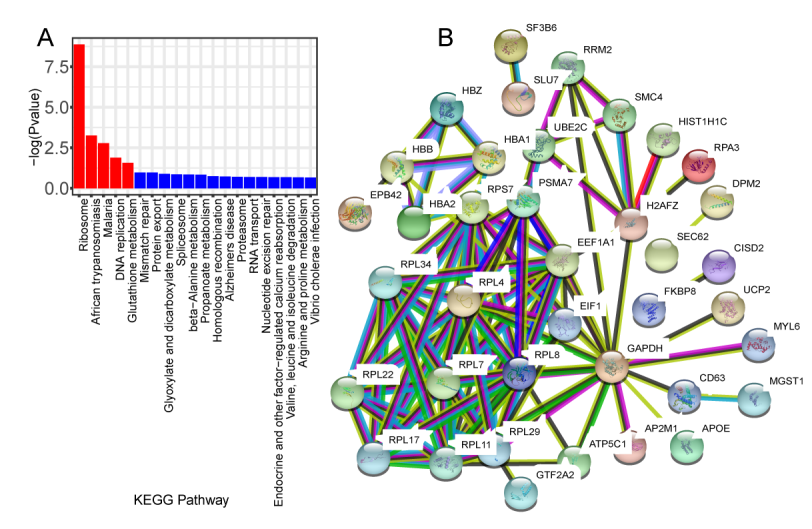


**Figure S12**. Analysis of enriched KEGG pathways (A), and protein-protein interaction networks (B) among the genes of the module-blue depicted in the 8 PCW liver.


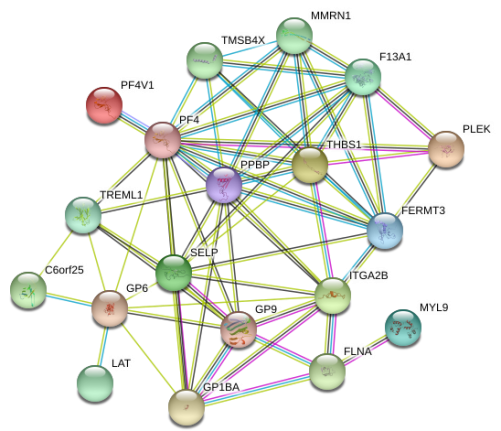


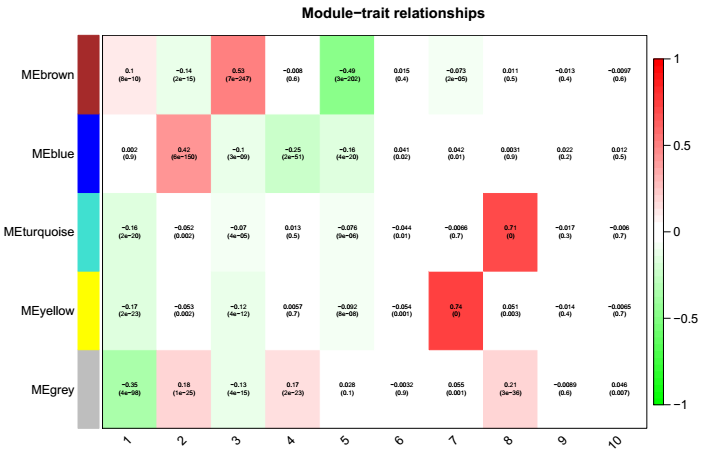


CA

AA


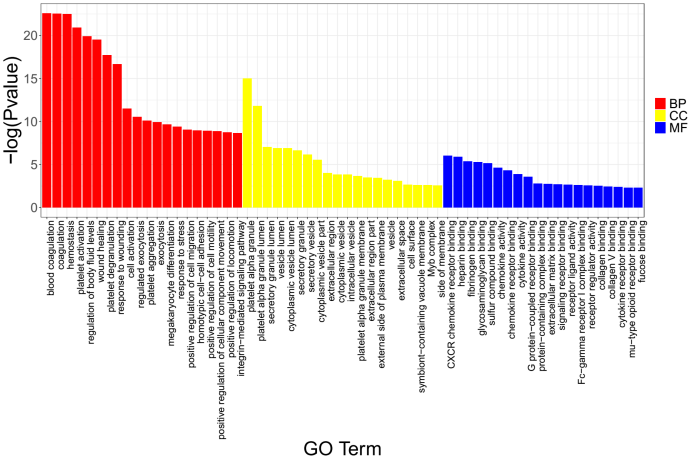


BA

DA


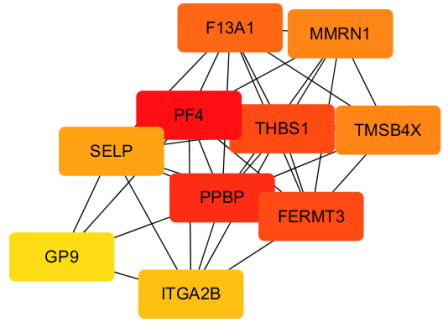


**Figure S13**. Expression pattern and coexpression networks of embryonic liver development Genes. (A) The heatmap shows the Spearman correlation of coexpression modules in the 17 PCW liver. (B-D) Analysis of enriched Gene Ontology terms (B) and protein-protein interaction networks (C), and identification of hub gene among the genes of the module-yellow (D).


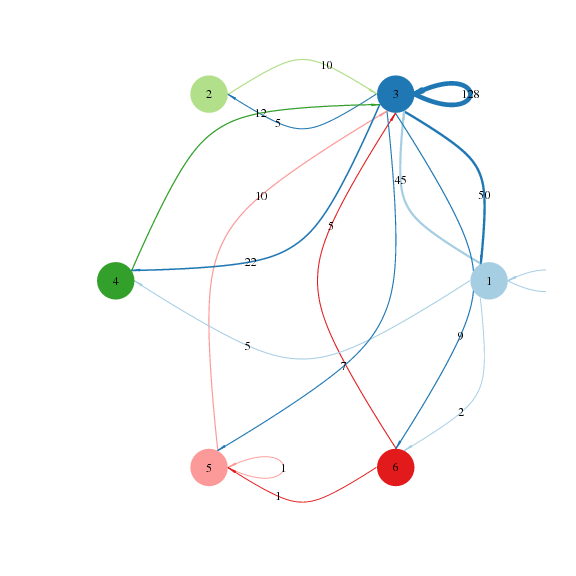


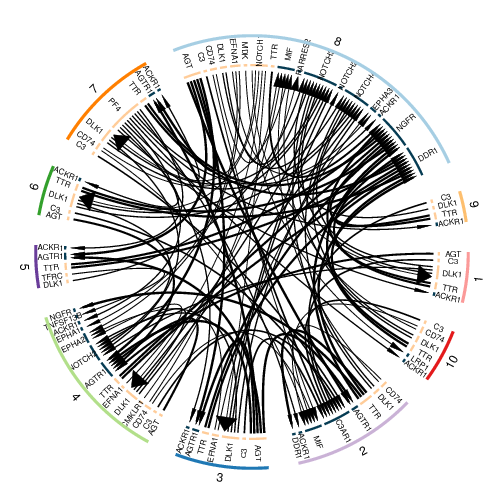


AA

BA

CA


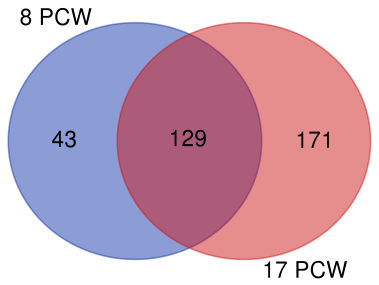


**Figure S14**. Cellular communication analysis of the cell clusters in the 8 PCW liver (A) and 17 PCW (B) liver. PCW, postconception weeks. Venn diagram showing the overlaps of ligand-receptor pairs in the 8 PCW and 17 PCW liver (C).
